# Effects of Targeted Radionuclide Therapy on Cancer Cells Beyond the Ablative Radiation Dose

**DOI:** 10.3390/ijms26146968

**Published:** 2025-07-20

**Authors:** Guillermina Ferro-Flores, Erika Azorín-Vega, Blanca Ocampo-García, Myrna Luna-Gutiérrez, Pedro Cruz-Nova, Laura Meléndez-Alafort

**Affiliations:** 1 Department of Radioactive Materials, Instituto Nacional de Investigaciones Nucleares, Ocoyoacac 52750, Mexico; guillermina.ferro@inin.gob.mx (G.F.-F.); erica.azorin@inin.gob.mx (E.A.-V.); blanca.ocampo@inin.gob.mx (B.O.-G.); myrna.luna@inin.gob.mx (M.L.-G.); 2 Immunology and Molecular Oncology Unit, Veneto Institute of Oncology IOV-IRCCS, Via Gattamelata 64, 35138 Padova, Italy

**Keywords:** targeted radionuclide therapy, therapeutic radiopharmaceuticals, immunotherapy, tumor microenvironment

## Abstract

Targeted radionuclide therapy (TRT) utilizes radiopharmaceuticals to deliver radiation directly to cancer cells while sparing healthy tissues. Beyond the absorbed dose of ablative radiation, TRT induces non-targeted effects (NTEs) that significantly enhance its therapeutic efficacy. These effects include radiation-induced bystander effects (RIBEs), abscopal effects (AEs), radiation-induced genomic instability (RIGI), and adaptive responses, which collectively influence the behavior of cancer cells and the tumor microenvironment (TME). TRT also modulates immune responses, promoting immune-mediated cell death and enhancing the efficacy of combination therapies, such as the use of immune checkpoint inhibitors. The molecular mechanisms underlying TRT involve DNA damage, oxidative stress, and apoptosis, with repair pathways like homologous recombination (HR) and non-homologous end joining (NHEJ) playing critical roles. However, challenges such as tumor heterogeneity, hypoxia, and radioresistance limit the effectiveness of this approach. Advances in theranostics, which integrate diagnostic imaging with TRT, have enabled personalized treatment approaches, while artificial intelligence and improved dosimetry offer potential for treatment optimization. Despite the significant survival benefits of TRT in prostate cancer and neuroendocrine tumors, 30–40% of patients remain unresponsive, which highlights the need for further research into molecular pathways, long-term effects, and combined therapies. This review outlines the dual mechanisms of TRT, direct toxicity and NTEs, and discusses strategies to enhance its efficacy and expand its use in oncology.

## 1. Introduction

Targeted radionuclide therapy (TRT) represents a transformative approach in cancer treatment that utilizes radiopharmaceuticals to deliver radiation directly to malignant cells while minimizing the damage to surrounding healthy tissues. This precision-based therapy has gained significant attention due to its ability to achieve localized cytotoxic effects. Beyond the direct cytotoxicity of absorbed doses of radiation, TRT triggers non-targeted effects, including systemic immune responses, radiation-induced bystander effects (RIBEs), radiation-induced cohort effects (RICEs), and radiation-induced genomic instability (RIGI) [1,2,3,4,5,6,7,8,9,10,11,12]. These mechanisms collectively amplify the therapeutic efficacy of TRT, offering new avenues for cancer management.

Despite its promise, TRT faces several challenges that limit its widespread application and effectiveness. These include the heterogeneous expression of molecular targets within tumors, clonal selection phenomena, and variable absorbed radiation dose distribution due to dynamic tumor perfusion and retention. Additionally, the tumor microenvironment (TME) plays a critical role in modulating TRT outcomes, often presenting conditions such as hypoxia, immune suppression, and stromal cell activation that reduce the treatment’s efficacy. Cancer cells further complicate therapy by evading cell death pathways, such as apoptosis, and developing resistance mechanisms [8,9,10,11,12].

Optimizing TRT requires a deeper understanding of the molecular pathways that govern cancer-cell responses, the interplay between TRT and TME, and the long-term consequences of non-targeted effects. Furthermore, combining TRT with immunotherapy has shown potential to enhance therapeutic outcomes, but this approach introduces complexities related to balancing efficacy and toxicity [8,9,10,11,12]. Addressing these challenges requires multidisciplinary efforts to refine treatment protocols, improve dosimetry, and develop novel radiopharmaceuticals that target both cytoprotective and cytotoxic pathways.

This review aims to explore the molecular mechanisms underlying TRT, analyze its effects on the TME, evaluate its long-term consequences on cancer cells and adjacent healthy tissues, and discuss current challenges in optimizing TRT for clinical applications. By advancing our understanding of these aspects, we can pave the way for more effective and personalized cancer treatments.

## 2. Current General Aspects of Targeted Radionuclide Therapy

### 2.1. Radiopharmaceuticals as the Basis of Targeted Radionuclide Therapy

Targeted radiopharmaceuticals are pharmaceutically formulated specific recognition molecules that contain alpha- or beta-particle-emitting radionuclides and are used for therapeutic purposes. Radiopharmaceuticals represent a rapidly evolving modality in targeted cancer radiotherapy. Their ability to deliver radiation with high specificity to malignant tissues, combined with advancements in theranostics, immunotherapy, and personalized treatment approaches, positions them as a critical component in the future of oncology [13,14].

The radiopharmaceuticals used in TRT have key components such as a targeting biomolecule that ensures the specific delivery of the radionuclide to the target tissue, a linker that modifies the excretion kinetics of the radiotracer, and a bifunctional agent or moiety site that facilitates radioactive labeling with radionuclides, in addition to the radionuclide itself, which emits ionizing radiation to destroy cancer cells. Some of the benefits of TRT radiopharmaceuticals include their ability to target malignant cells specifically, a lower likelihood of side effects compared to traditional chemotherapy or radiotherapy, and their non-invasive nature, which reduces long-term toxicities and complications [13].

Among the targeting biomolecules, the most relevant to therapeutic applications are the conjugates based on peptides and antibodies. Peptides are the most successful target-specific molecules used in TRT since they can quickly penetrate tumors with high specificity. Targeted peptide sequences can be engineered to identify specific receptors on cell surfaces and different components of the tumor microenvironment, providing numerous options for selecting therapeutic targets. Radiolabeled peptide therapies, have demonstrated clinical efficacy in the treatment of neuroendocrine tumors (targeting somatostatin receptors, SSTR) and metastatic castration-resistant prostate cancer (mCRPC) (targeting prostate-specific membrane antigen, PSMA). Notable examples include ^177^Lu-DOTA-TATE (bifunctional chelator: 1,4,7,10-tetraazacyclododecane-1,4,7,10-tetraacetic acid = DOTA; Tyr^3^-octreotate = TATE), ^177^Lu-DOTA-TOC (Tyr^3^-octreotide = TOC), ^90^Y-DOTA-TOC, ^177^Lu-PSMA-617, ^177^Lu-iPSMA, and ^177^Lu-PSMA-I&T [15,16,17,18,19].

According to the FDA, 13 radiopharmaceuticals have been approved for therapeutic use and 54 for diagnostic purposes [16,17]. A select group of radiopharmaceuticals has gained prominence in global therapeutic applications; among these are Lutathera^TM^ (^177^Lu-DOTA-TATE), Pluvicto^TM^ (^177^Lu-PSMA-617), and Radium-223 Dichloride (Xofigo^TM^), the latter of which is also used in the treatment of mCRPC [19].

Antibody-based radiopharmaceuticals selectively bind to cell-surface antigens that are overexpressed on cancer cells. For instance, ^90^Y-anti-CD20 and ^213^Bi-anti-CD33 bind to CD20 and CD33, respectively, in lymphomas and leukemias, while ^177^Lu-trastuzumab binds to the HER2 receptor in breast cancer. In this context, yttrium-90 ibritumomab tiuxetan (Zevalin^TM^), a ^90^Y-labeled anti-CD20 monoclonal antibody, has been approved by the FDA since 2002, making it the first radioimmunotherapy for the treatment of non-Hodgkin lymphoma [17]. However, despite their high affinity and specificity, the main limitations of antibody-based radiopharmaceuticals are immunogenicity and prolonged blood circulation times, which can result in slow tumor penetration. Nonetheless, the engineering of nanoantibodies and single-chain variable fragments (scFvs)labeled with therapeutic radionuclides exemplifies a progression in antibody pharmacokinetic properties and renders them viable candidates for use in the field of nuclear medicine [16,17,18,19,20].

Likewise, targeted alpha therapy (TAT) that employs ^225^Ac and ^211^At holds considerable promise as a localized treatment for cancer. The utilization of peptides and antibodies labeled with alpha emitters has demonstrated encouraging clinical outcomes in the treatment of patients afflicted with metastatic cancers. This approach holds particular significance for cases that have exhibited resistance to TRT that employs beta emitters, such as ^177^Lu and ^90^Y [21,22]. The FDA continues to approve new radiopharmaceuticals, including those with expanded indications. Therefore, the global radiopharmaceutical sector is projected to experience significant growth in the coming years.

### 2.2. Theranostics in Nuclear Medicine

Theranostics, in nuclear medicine, is an approach that combines diagnostic imaging capabilities with TRT, enabling personalized treatment in oncological diseases. This method leverages the unique properties of radionuclides, which emit radiation for both therapeutic and diagnostic purposes, and thereby contributes to the precise management of diseases. The integration of diagnostic and therapeutic radionuclides conjugated to the same molecular probe for targeting specific disease biomarkers is at the core of this approach [23].

Although the theranostics concept has its roots in the 1940s, with the use of radioactive iodine to treat thyroid diseases, it has undergone significant evolution over the decades. Recent advancements in the development of theranostic pairs are expanding the applications of oncological TRT and enhancing the utilization of molecular imaging for treatment guidance. Globally, the most used theranostic radionuclide pairs in clinical practice are ^68^Ga (PET: positron emission tomography)/^177^Lu (beta therapy), ^68^Ga (PET)/^225^Ac (alpha therapy), ^64^Cu (PET)/^177^Lu (beta therapy), ^64^Cu (PET)/^225^Ac (alpha therapy), ^18^F(PET)/^177^Lu (beta therapy), and ^99m^Tc (SPECT: single-photon emission computed tomography)/^177^Lu (beta therapy). Likewise, the isotopic radionuclide pairs ^64^Cu/^67^Cu, ^152^Tb/^161^Tb, ^83^Sr/^89^Sr, ^124^I/^131^I, ^203^Pb/^212^Pb, ^43/44^Sc/^47^Sc, ^86^Y/^90^Y, and ^110^In/^111^In have shown promising potential in theranostics. In current medical practice, diagnostic radiopharmaceuticals such as ^68^Ga-PSMA-11, ^99m^Tc-iPSMA, ^99m^Tc-PSMA-I&T, and ^18^F-PSMA-1007 are used to diagnose prostate cancer. Meanwhile, ^68^Ga-DOTA-TATE, ^68^Ga-DOTA-TOC, ^64^Cu-NOTA-TOC, ^99m^Tc-TOC, and ^18^F-TOC are used to diagnose neuroendocrine tumors. As was previously mentioned, the most used therapeutic radiopharmaceutical pairs are ^177^Lu-DOTA-iPSMA, ^177^Lu-PSMA-617, ^177^Lu-PSMA-I&T, ^225^Ac-PSMA-617, ^177^Lu-DOTA-TATE, and ^177^Lu-DOTA-TOC [23,24,25].

The benefits of theranostics include the ability to tailor treatment plans to individual patient characteristics and tumor biology, the potential to target both primary tumors and metastatic lesions, and a reduction in side effects compared to traditional radio- and chemotherapy [24].

It is important to note that the list of potential theranostic radiopharmaceuticals is extensive. Recently, radiopharmaceuticals that target the tumor microenvironment have been demonstrated to be viable options for use in routine clinical settings. Examples include ^99m^Tc-iFAP (SPECT), ^68^Ga-FAPI-04 (PET), and ^177^Lu-FAP-2286 (beta therapy), which target the fibroblast activation protein (FAP), and ^99m^Tc-iPD-L1 (SPECT), ^68^Ga-WL12 (PET), and ^177^Lu-iPD-L1 (beta therapy), which target the programmed cell death protein 1 (PD-1) ()/programmed death ligand 1 (PD-L1) () pathway [8,24,26,27,28,29]. Combining TRT with immunotherapy has shown the potential to enhance the efficacy of treatment beyond the cytotoxic effects of the absorbed radiation dose. In particular, the modulatory effects of targeted radiopharmaceuticals on immune checkpoints (ICs), such as PD-1, PD-L1, and cytotoxic T-lymphocyte antigen 4 (CTLA-4), are being actively studied (Table 1).

Personalized dosimetry is crucial in theranostic applications for optimizing the treatment efficacy and minimizing toxicity. This approach involves estimating the doses of radiation absorbed by organs and tumors based on individual patient characteristics. Although personalized dosimetry faces technical, methodological, and practical challenges, the use of commercial computer programs such as PLANET Dose, OLINDA/EXM, and Torch facilitates dosimetry calculations in clinical applications [52,53]. The future of theranostics holds great promise due to technological advances, including the integration of artificial intelligence to enhance diagnostic accuracy and treatment planning [54,55,56,57].

### 2.3. Recent Clinical Outcomes of Targeted Radionuclide Therapy

As mentioned earlier, targeted radiopharmaceuticals have demonstrated significant improvements in the patient survival rates for various cancers, particularly neuroendocrine and prostate cancers [58,59]. Generally, all radiopharmaceuticals show significant survival times and response rates in their respective patient populations. ^177^Lu-DOTA-TATE and ^177^Lu-DOTA-TOC are particularly effective for neuroendocrine tumors, while ^177^Lu-PSMA-617, ^177^Lu-DOTA-iPSMA, and ^177^Lu-PSMA I&T show significant efficacy in treating mCRPC (Table 2).

## 3. Immediate Molecular and Cellular Responses to Ablative Radiation Dose

The initial molecular and cellular responses to targeted ablative radiation doses of radiopharmaceutical radiation entail the activation of DNA damage recognition factors, which subsequently trigger signaling cascades. These cascades regulate the expression and activity of genes and proteins that are responsible for cell-cycle arrest, DNA repair, accelerated senescence, and apoptosis. The nature of the response is contingent upon the rate of administration. It has been demonstrated that low-dose-rate exposures promote apoptosis, whereas high-dose-rate exposures primarily induce necrosis [68,69,70].

### 3.1. DNA Direct and Indirect Damage Induced by Radiation

The early molecular and cellular effects of doses of targeted ablative radiation dose with radiopharmaceuticals involve the activation of DNA damage recognition factors, which initiate signaling cascades. Such cascades modulate the expression and activity of genes and proteins that are responsible for cell-cycle arrest, DNA-repair, accelerated senescence, and apoptosis. The responses vary with the dose rate, as low-dose-rate exposures promote apoptosis, while high-dose-rate exposures primarily induce necrosis [68,69,70].

After exposure to ionizing radiation, direct and indirect DNA damage can occur. Direct damage is caused by ionization, while indirect damage is mediated by reactive oxygen species (ROS) or reactive nitrogen species (RNS), which are primarily generated through the interaction of radiation with cellular water molecules. These reactive species can diffuse from the site of formation and interact with any molecules in their path.

The type and extent of DNA damage depend on the characteristics of the radiopharmaceutical. Particularly, they depend on the emitted radiation and the proximity of the radionuclide to the DNA. The types of DNA damage are classified as follows:

(a) Single-strand breaks (SSBs): SSBs occur when the phosphate backbone of a DNA strand is broken. SSBs are generally less lethal than double-strand breaks, but they can lead to mutations if they are not repaired correctly [71,72,73]. The single-strand break repair pathway involves a group of complex proteins, such as X-ray repair cross-complementing protein 1 (XRCC1).

(b) Double-strand breaks (DSBs): Although less frequent, these types of DNA damage are highly toxic and typically result from ionizing radiation. DSBs are challenging to repair, and improper repair can lead to mutations or cell death. DSBs are typically visualized using immunofluorescence, a technique that employs antibodies that are directed against proteins that are formed in response to DNA damage, such as γ-H2AX and 53BP1. These antibodies identify and highlight impaired structures within a sample [74,75,76]. ^177^Lu radiopharmaceuticals primarily induce DSBs due to the emission of beta particles, which directly damage DNA, which results in alterations to the cell cycle, the accumulation of micronuclei, and cell death. A direct comparison of between DSBs induced by ^177^Lu-DOTA-TATE and ^177^Lu-PSMA-617 revealed that ^177^Lu-PSMA-617 induced significantly more DSBs in tumor cells due to a higher dose being absorbed in tumors. However, this difference cannot be explained solely by the absorbed radiation dose; other factors, such as the tumor biology, receptor density, and radiation quality, must also be considered [77]. The effects of ^177^Lu induce the activation of DNA-damage response markers that are involved in DSB repair mechanisms, such as non-homologous end joining (NHEJ), homologous recombination (HR), and alternative end-joining [70,78,79,80].

DNA damage has been studied in the blood lymphocytes of patients who were treated with ^177^Lu-DOTA-TATE or ^177^Lu-DOTA-TOC. The number DSBs was quantified [79]. It was observed that the average number of radiation-induced foci (RIF) per cell increased over the first 5 h after radionuclide administration, as a function of the dose absorbed by the blood. At later time points, the number of RIF decreased, which indicated the DNA repair phase. The mathematical model describing this behavior can be observed in Equation (1).

(1) $$N\left(t\right)=(a+m\cdot b\cdot {\bar{D}}_{blood}\;\left(t\right)).(k\cdot e^{-\lambda t}+(1-k\cdot e^{-vt})$$

*N(t)* = number of radiation-induced foci (RIF) at time *t*;

*m* = parameter for the variability in patient dosimetry, used for the in vitro calibration;

*a*, *b* = constants for in vitro calibration curve that represent the RIF number per cell as a function of the mean time-dependent absorbed dose ($\bar{D}$).

The pattern of DSB induction and repair observed when using ^177^Lu-PSMA was analogous to that of ^177^Lu-DOTA-TOC or ^177^Lu-DOTA-TATE [78]. However, a subsequent study demonstrated that, in patients with ^177^Lu-PSMA, the RIF produced was higher than that produced by ^177^Lu-DOTA-TOC when the two were compared 5 min after injection [81].

For radiopharmaceuticals based on alpha particles, simulation using TOPAS-nBio showed that the yield of DSBs per unit of dose correlates with the dose-mean lineal energy and specific energy, which indicates that more compacted DNA experiences a higher frequency of DSBs compared to unfolded DNA due to the stochastic energy deposition from alpha particles [82]. Alpha particles interact directly with DNA as well as indirect effects mediated by ROS production. Several studies have reported that exposure to alpha particles induces chromosomal instability, which can be inherited by progeny cells [83]. The ex vivo irradiation of blood with the only FDA-approved alpha radiopharmaceutical, ^223^RaCl_2_, resulted in the detection of DNA DSBs in peripheral blood mononuclear cells. The induction of these DSBs exhibited a dose-dependent relationship, with an observed increase at 25 mGy, 50 mGy, and 100 mGy. Concurrently, the rate of repair was higher at lower doses: 0.24/h at 25 mGy, 0.16/h at 50 mGy, and 0.13/h at 100 mGy. After 24 h, a fraction of the damage remained unrepaired [84]. The short-term exposure to ^223^RaCl_2_ resulted in an increased number of DSBs, as observed by 53BP1 foci, which led to reduced cell survival and cell-cycle arrest. The DNA repair mechanisms induced by ^223^Ra involve homologous recombination and non-homologous end joining to maintain genomic stability [85]. Targeted alpha therapy clinical studies using radiopharmaceuticals based on ^211^At, ^225^Ac, and ^212^Pb are currently in progress (https://clinicaltrials.gov/).

Auger electrons enable the delivery of high doses of radiation within 10 nm. Consequently, molecules near the Auger cascade are damaged by both the direct effect of electron irradiation and the indirect effects caused by ROS. The decay of ^111^In, ^123^I, ^125^I, and others exhibits high radiotoxicity when they decay within the DNA [86].

### 3.2. DNA Damage Mediated by ROS and RNS

ROS are subproducts of O_2_ reduction in the mitochondrial respiratory chain. They comprise superoxide anion free radicals (O_2_^•−^), hydroxyl radicals (OH), hydrogen peroxide (H_2_O_2_), lipid peroxides (LOOH), singlet oxygen (^1^O_2_), hypochlorous acid (HOCl), chloramines (RNHCl), and ozone (O_3_). The overproduction of ROS disrupts homeostasis and leads to oxidative stress [87,88].

While ROS induces DNA damage and apoptosis in cancer cells, they can also damage normal cells. ROS generated by a mitochondria-targeted titanium dioxide-gold nano-radiosensitizer produced mitochondrial dysfunction and irreversible apoptosis. The ROS accumulation enhanced the efficacy of radiation therapy. The nanosensitizer reduced the X-ray dose and the number of treatments [89].

In the context of radiotherapy, ROS-mediated DNA damage can be used to enhance the efficacy of treatment. Oxidative stress can induce apoptosis, autophagy, and ferroptosis. It is also implicated in the tumor immune response, mainly due to influencing the release of tumor-associated antigens, regulating immune cell infiltration and differentiation, and affecting the expression of immune checkpoints [88].

^177^Lu-PSMA-617 induces ROS generation through the emission of beta particles, leading to DSBs and oxidative stress [80].

### 3.3. Molecular Pathways Involved in DNA Damage as a Response to Ablative Radiation Doses in Tumors

**Figure 1:**
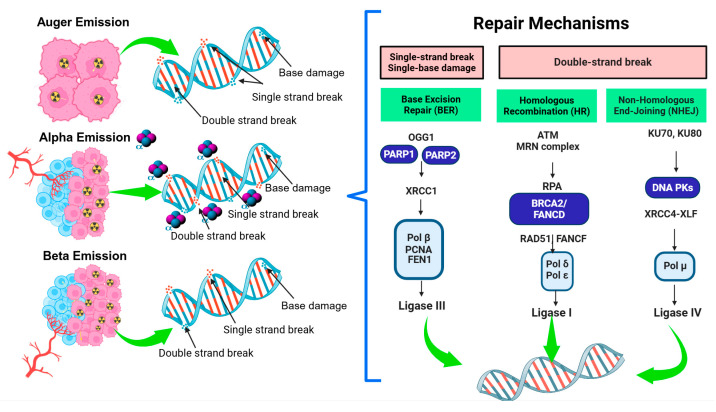
Molecular pathways involved in DNA damage resulting from ablative radiation doses in tumors. Proteins involved in repair pathways: 8-oxoguanine (OGG1), XRCC1 (X-ray cross-complementing protein 1), DNA polymerases β, δ, ε (pol β, δ, and ε), Flap endonuclease 1 (FEN1), proliferating cell nuclear antigen (PCNA), PARP (which catalyzes the formation of poly-ADP-ribose, PAR), XRCC4 (X-ray cross-complementing protein 4); BRCA2 (a tumor suppressor protein), and XLF (XRCC4-like factor). The Ku70/80 heterodimer is the first protein to bind to DSBs. Ligases seal the nick in the DNA backbone.

The use of ablative radiation doses aims to destroy cancer cells while minimizing the damage to healthy tissues. The efficacy of this response depends on the ability of the tumor to respond and repair DNA damage, which determines whether cells survive or die as a result of radiation-induced damage. As mentioned above, the two main repair mechanisms that detect and repair DSBs are NHEJ and HR [73] (Figure 1).

The NHEJ pathway is prone to errors but remains active throughout the cell cycle. It is the primary mechanism for repairing DSBs in mammalian cells, especially in the absence of homologous recombination. Key proteins involved in NHEJ include the Ku70/80 heterodimer, DNA-dependent protein kinase complex (DNA-PK), MRE11-RAD50-NBS1 complex, and ligase IV. Studies indicate that the inhibition of DNA-PK increases the sensitivity of tumor cells to X-ray irradiation or proton beam therapy in head and neck squamous cell carcinoma [73,90].

HR is an accurate repair mechanism that relies on homologous sequences to repair base substitution mutations and base insertion/deletion mutations. This pathway is active during the S and G2 phases of the cell cycle. Essential proteins in HR include BRCA1, BRCA2, and RAD5. Deficiencies in HR, such as those observed in BRCA-mutated cancers, can lead to increased radiosensitivity due to the inability to repair radiation-induced DSBs (Figure 1).

Recent studies have highlighted the significant role of nicotinamide N-methyltransferase (NNMT) in cancer stem cell radioresistance and its potential as a therapeutic target. Cancer stem cells, which demonstrate greater resistance to radiation than normal stem cells, often exhibit the overexpression of NNMT, which contributes to enhanced DNA repair and tumor recurrence following radiotherapy [91,92]. This enzyme, which methylates nicotinamide to form 1-methylnicotinamide (MNA), is implicated in various disease contexts, including cancer, diabetes, and obesity. Novel macrocyclic peptides have been identified as potent allosteric inhibitors of NNMT [93]. Further advancements include the development of bisubstrate NNMT inhibitors with electron-deficient aromatics and optimized linkers, which has led to the development of compounds with IC50 values as potent as 3.7 nM, which are among the most effective reported to date [94]. However, translating this potency to cellular activity has been challenging; prodrug strategies, such as the use of isopropyl ester derivatives, have significantly improved the cell permeability and demonstrated effective NNMT inhibition in live cells [95]. Together, these findings underscore the importance of NNMT in cancer biology and the promise of targeted NNMT inhibitors in overcoming resistance mechanisms.

### 3.4. Cellular Signaling and Apoptosis

The ataxia telangiectasia mutated (ATM) and ataxia telangiectasia and Rad3-related (ATR) kinases serve as the primary activators of the DNA damage response (DDR). The DNA repair mechanisms that are responsible for maintaining genomic integrity when damage is caused by ionizing radiation include base excision repair (BER), nucleotide excision repair, mismatch repair (MMR), double-strand break repair (DSBR), HR, NHEJ, translesion synthesis, cellular signaling, and apoptosis. Radiation exposure triggers cellular signaling pathways, including the epidermal growth factor receptor (EGFR), which modulates cell survival and apoptosis. The immediate cellular response involves activating signal transduction cascades, which can lead to either cytoprotective or cytotoxic outcomes, depending on the context and the extent of damage. [96]. Furthermore, the activation of pathways such as the ATM-tumor protein 53 (TP53), mitogen-activated protein kinasa (MAPK), and nuclear factor kappa B (NF-κB) pathways results in altered gene expression and cellular responses, influencing cell survival or death.

### 3.5. Apoptosis and Cell Death

Radiopharmaceuticals induce apoptosis through both intrinsic and extrinsic pathways. Intrinsic apoptosis is mediated by mitochondrial pathways and triggered by cellular signals of internal stress, such as DNA damage, hypoxia, and endoplasmic reticulum stress. These signals depend on BCL2-associated X (BAX) and BCL2 homologous antagonist killer (BAK), which are responsible for releasing cytochrome C. Extrinsic apoptosis involves the activation of the immune system by external cellular stimuli [97]. Ablative radiation doses trigger distinct waves of cell death depending on the cell-cycle phase at the time of irradiation. For example, cells irradiated in the S/G2 phase undergo mitotic death, whereas those irradiated in the G1 phase experience delayed cell death associated with chromosome segregation errors and pro-inflammatory responses.

Several studies have evaluated apoptosis by using therapeutic radiopharmaceuticals as a tool to monitor the efficacy of cancer treatment and detect radiopharmaceutical-induced cell damage to the tumor (Table 3).

### 3.6. Cell-Cycle Arrest and Checkpoint Activation

Radiation-induced DNA damage activates cell-cycle checkpoints, which allows for time for repair before cell division occurs. However, the activation of these checkpoints can contribute to radioresistance. Inhibitors that target DDR proteins, such as ATR and ATM, abrogate these checkpoints, forcing cells to proceed through the cell cycle with unresolved DNA damage. This behavior leads to mitotic catastrophe and cell death [107,108].

## 4. Tumor Microenvironment Changes: Immune-Mediated Cell Death Induced by Targeted Radionuclide Therapy

Antigen-presenting cells (APCs) (myeloid cells) are involved in diverse immunological processes, trafficking through the circulatory and lymphatic systems to be subsequently recruited to sites of tissue injury or infection [109].

APCs includes macrophage heterogeneity and dendritic cells (DCs). DCs exhibit phenotypic, transcriptional, and functional differences across different tissues. This population encompasses conventional dendritic cells, plasmacytoid DCs, unconventional type 3 DCs, and monocyte-derived DCs. Conventional DCs are further classified into type 1 and type 2 subsets, each of which is specialized in antigen uptake and presentation. Conventional type 1 DCs primarily present antigens to CD8^+^ T lymphocytes via the major histocompatibility complex class I (MHC-I).

In contrast, conventional type 2 DCs are mainly involved in presenting antigens to CD4^+^ T lymphocytes through MHC class II (MHC-II) [110]. Macrophages are another type of myeloid cell that is characterized by a high level of phagocytosis [111]. Macrophages are key effectors of the innate immune system, playing both direct and indirect roles in host defense against pathogens. They are equipped to recognize signals associated with pathogen invasion or tissue damage. Most macrophages express a wide array of pattern recognition receptors (PRRs), including Toll-like receptors, C-type lectins, and cytosolic sensors such as nucleotide-binding oligomerization domain receptors [112].

APCs detect tumor-derived danger signals, infiltrate the tumor microenvironment, and capture tumor-associated antigens. Upon activation, APCs migrate to the draining lymph nodes, where they prime tumor-specific T lymphocytes. These activated T cells subsequently migrate to the tumor site to mediate cytotoxic responses and promote tumor clearance [113]. DCs are critical regulators of the response to immune checkpoint blockade and other cancer immunotherapies. This behavior is evidenced by the association between the presence of intratumoral DCs or the expression of specific DC-related transcriptional signatures and an inflamed T-cell phenotype, as well as enhanced CD8^+^ T-cell infiltration [114]. In addition, the tumor microenvironment comprises various immune cells, including tumor-associated macrophages (TAMs) with an M2-like (pro-tumoral and anti-inflammatory) phenotype, tumor-associated neutrophils, myeloid-derived suppressor cells, mast cells, and natural killer (NK) cells. These cells secrete a broad spectrum of factors, including chemokines, cytokines, and enzymes, which collectively modulate tumor progression, immune evasion, and tissue remodeling [115].

Somatostatin analogs, such as ^177^Lu-DOTA-TATE and ^177^Lu-DOTA-TOC, are among the most widely used agents in nuclear medicine. Treatment with ^177^Lu-DOTA-TATE in human bronchial neuroendocrine tumors enhances the infiltration of CD86^+^ APCs into the tumor microenvironment. Additionally, a significant increase in FasL^+^CD49b^+^ NK cells—indicative of activated cytotoxic NK subsets—was observed. However, an analysis of Fas receptor expression, a marker associated with radiosensitivity, revealed no significant differences compared to untreated controls [116]. These findings suggest that combining ^177^Lu-DOTA-TATE therapy with radiosensitizing agents, such as selected immunotherapies, may improve therapeutic efficacy.

**Figure 2:**
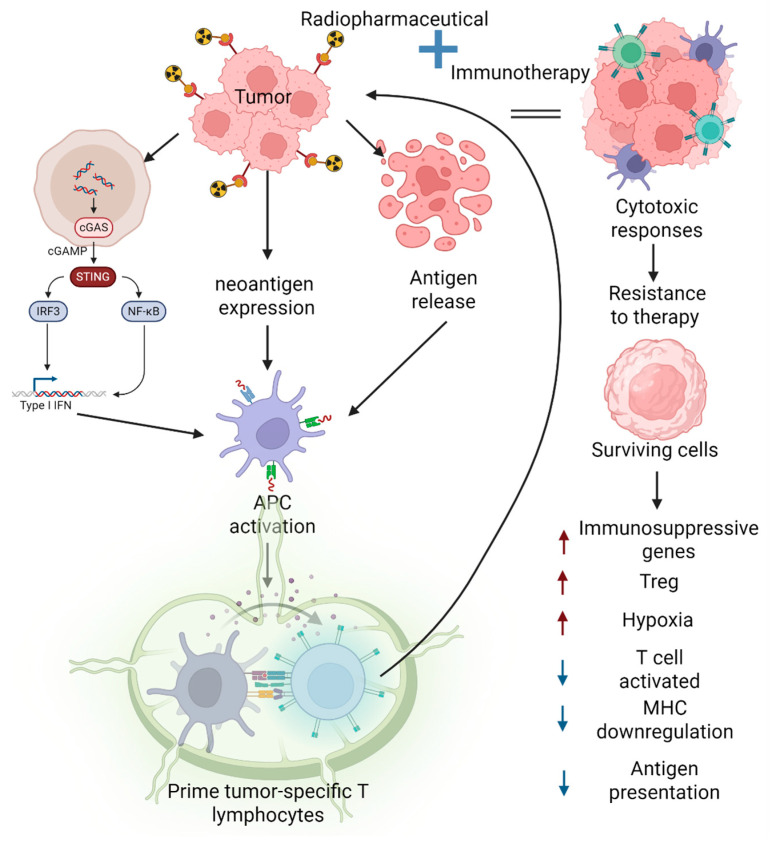
Immune-mediated cell death induced by targeted radionuclide therapy.

Another key regulator of the immune system within the tumor microenvironment is the population of cancer-associated fibroblasts (CAFs), which are increasingly recognized as potential targets for cancer therapy. The inhibition of transforming growth factor-beta (TGF-β) signaling and NOX4 activity has demonstrated efficacy in modulating CAFs function, promoting T-cell infiltration, and enhancing the response to immunotherapy in preclinical models [117]. For instance, in a murine fibrosarcoma model (MCA205) stably expressing FAP, treatment with 60 MBq of the radiopharmaceutical ^177^Lu-FAP-2287 in combination with an anti-PD-1 immune checkpoint inhibitor resulted in a greater infiltration of CD45^+^ leukocytes compared to monotherapy. The proposed mechanism involves radiation-induced DNA damage, which activates the cyclic GMP–AMP synthase-stimulator of interferon genes (cGAS–STING) pathway and subsequently promotes the expression of type I interferons, such as IFN-γ, along with chemokines CCL5 and CXCL10, facilitating the recruitment of CD8^+^ T cells. As a result, the combination therapy was associated with the increased infiltration of CD8^+^ T lymphocytes, CD86^+^-activated monocytic myeloid-derived suppressor cells, and TAMs, ultimately contributing to a robust anti-tumor immune response [8]. Cytosolic DNA serves as a key trigger of the inflammatory response following radiotherapy. This treatment induces both direct and indirect damage to nuclear and mitochondrial DNA, resulting in the generation of DNA fragments that accumulate in the nucleus and cytoplasm. These fragments are sensed by the cGAS, which activates the STING signaling pathway. The activation of this pathway leads to the production of type I interferons (IFNs), a response that is essential for the recruitment and activation of cytotoxic CD8^+^ T lymphocytes after radiotherapy (Figure 2) [118].

In a related study, treatment with the combination therapy of ^177^Lu-DOTA-2P(FAPI)_2_ plus anti-PD-L1 monoclonal antibody resulted in a significant modulation of the tumor immune microenvironment, which was characterized by an increased infiltration of intratumoral T lymphocytes. Notably, the most prominent expansion was observed in effector T-cell subsets, which are associated with enhanced therapeutic efficacy and the establishment of durable immune memory. Furthermore, the results revealed an elevated proportion of NK cells in the tumor microenvironment following treatment with ^177^Lu-DOTA-2P(FAPI)_2_ alone or in combination with anti-PD-L1. This alteration was accompanied by an upregulation of the tumor-associated FASLG signaling pathway and a concomitant downregulation of the tumor-promoting SPP1 pathway [50]. Radiation exerts several interdependent immunomodulatory effects, including enhancement of the maturation and effector function of tumor-infiltrating lymphocytes, such as T cells and NKcells, through the depletion of immunosuppressive cells within the tumor microenvironment. For instance, the combination of a PD-L1 inhibitor (iPD-L1) with radiotherapy using ^177^Lu-labeled iPD-L1 (^177^Lu-iPD-L1) significantly reduced the viability of PD-L1–positive cancer cells. This treatment also promoted a robust immunomodulatory response that was characterized by an increase in activated macrophages and elevated expression levels of PD-L1, IL-10, and TGF-β. These changes collectively enhanced the therapeutic efficacy against PD-L1-expressing tumors in a murine model of triple-negative breast cancer [14].

Based on the hypothesis that activation of the adaptive immune system occurs through T-lymphocyte stimulation via antigen presentation, various strategies have been developed to enhance this process. Among them, low-dose radiotherapy (LDRT) has emerged as a promising approach, as it facilitates T-cell infiltration and improves the responsiveness of immunotherapy in an IFN-dependent manner. In a murine model of ovarian cancer, the combination of LDRT (1 Gy administered at weekly intervals) and immunotherapy was shown to promote the activation of a subset of DCs that express the NKG2D ligand RAE1. This behavior, in turn, has been shown to stimulate T-cells with cytotoxic effector characteristics. These T-cells include a subset that expresses NKG2D, an important costimulatory receptor that has been demonstrated to enhance the cytolytic function of CD8^+^ T cells and exhibit proliferative capacity [119]. Therefore, in patients with cervical cancer treated with combined chemo- and radiotherapy, an upregulation of MHC class II genes was observed in the epithelial cells post-treatment, which suggested enhanced immunogenicity. However, no accumulation of CD4^+^ or CD8^+^ T lymphocytes was detected three weeks after therapy. This discrepancy may be explained by the concurrent upregulation of immunosuppressive genes such as *LCN2* and *DEFB1* in epithelial cells that survived the treatment. These cells also exhibited a diminished antigen presentation profile and a reduced capacity to activate lymphocytes, indicating a potential mechanism of immune evasion that could contribute to treatment resistance (Figure 2) [120]. One of the principal mechanisms underlying resistance to combined radio/immunotherapy is the loss of antigen presentation, a strategy that is frequently employed by tumor cells to evade immune recognition and destruction. This impairment can hinder the maturation of tumor-specific T cells, rendering tumor cells ‘invisible’ to T-cell receptors (TCRs) and making them resistant to elimination by activated antitumor T cells. The loss or downregulation of MHC molecules, often resulting from mutations or the reduced expression of MHC genes, can significantly diminish the presentation of antigens [89] (Figure 2). Moreover, exposure to danger signals, such as tumor-derived double-stranded DNA (dsDNA), promotes DC maturation and the upregulation of key immunostimulatory molecules, including co-stimulatory ligands (CD80 and CD86) and cytokines such as interleukin-12 (IL-12), which supports the differentiation of cytotoxic T lymphocytes. However, regulatory T cells (Tregs) can suppress the stimulatory capacity of DCs within the tumor microenvironment, thereby impairing the CD8^+^ T-cell-mediated antitumor response. Furthermore, in lymph nodes, the microanatomical localization of DCs and CD8^+^ T cells during the priming phase plays a crucial role in shaping T-cell activation and effector differentiation (Figure 2) [121].

Accordingly, specific immune cell populations within the solid malignant tumor microenvironment may paradoxically promote tumor progression and metastasis, which highlights a dual role of the immune system in cancer. In particular, some immune cells that express very late antigen-4 (VLA-4) have been shown to contribute to metastasis by promoting immunosuppressive and proangiogenic signaling pathways. Notably, the radiopharmaceutical ^177^Lu-LLP2A, which exhibits a high affinity for VLA-4, has demonstrated potential in targeting these cells and may enhance therapeutic efficacy when used in combination with immunotherapy [30]. T-cell non-Hodgkin lymphoma serves as a valuable model for investigating cell-directed therapies that target immune cells. Given the selective uptake of alkylphospholipids by cancer cells, a radiolabeled alkylphosphocholine analog, ^90^Y-NM600, was developed. Treatment with this compound led to a significant increase in activated CD8^+^ T lymphocytes within the tumors. To investigate the contribution of immune memory, a re-challenge experiment was carried out: mice previously treated with 9.25 MBq of the radiopharmaceutical were re-inoculated with tumor cells. Remarkably, these mice did not develop tumors, which suggested the induction of long-lasting antitumor immunity. This response was associated with the increased production of IFN-γ by splenocytes from the treated animals [122].

Ionizing radiation can trigger the release of tumor antigens or induce the expression of neoantigens, and can thereby promote immunogenic cell death. Radiotherapy induces the expression of damage-associated molecular patterns (DAMPs), including the externalization of calreticulin and annexin A1, the release of high-mobility group box one protein (HMGB1) and ATP into the extracellular matrix, and the secretion of type I IFN. In a preclinical melanoma model, the interaction between the immune system and the radiopharmaceutical ^131^I-ICF01012, a melanin-targeting ligand, was evaluated. A combination of radiopharmaceuticals with anti-CTLA-4 immunotherapy significantly improved survival. Several factors may explain this enhanced efficacy: (i) the radiopharmaceutical does not target immune cells, (ii) a low dose (18.5 MBq) is used to minimize hematological toxicity, (iii) the compound exhibits rapid clearance, and (iv) it significantly upregulates the expression of annexin A1 and calreticulin on the tumor cell surface, thereby promoting immunogenic cell death [31] (Figure 2).

Furthermore, studies have shown that the alpha-emitting radiopharmaceutical radium-223 dichloride (^223^Ra) can induce immunogenic modulation in tumor cells that survive radiation exposure. When cancer cells from various origins, including the prostate, breast, and lung, were treated with sublethal doses of ^223^Ra (4, 10, and up to 40 Gy), enhanced lysis mediated by CD8^+^ cytotoxic T lymphocytes that are specific for tumor-associated antigens such as MUC-1, brachyury, and carcinoembryonic antigen (CEA) was observed. This increased cytotoxic activity was associated with the upregulated expression of MHC-I molecules and calreticulin, both of which are critical for effective antigen presentation. To optimize the therapeutic efficacy of ^223^Ra, it should be combined with immunotherapeutic strategies that are aimed at expanding tumor antigen-specific T-cell populations [123]. The mechanism of action of alpha-emitting radiopharmaceuticals extends beyond the induction of irreversible DNA double-strand breaks. For instance, the mesothelin-targeted thorium-227 conjugate (^227^Th-MSLN-TTC) has demonstrated immunomodulatory effects in MC38 colorectal cancer cells. Treatment with MSLN-TTC resulted in the upregulation of proinflammatory cytokines and chemokines, including interleukin-6 (IL-6), CCL20, and CXCL10, as well as genes associated with the STING signaling pathway.

Additionally, an increased expression of DAMPs was observed, which contributed to DC activation. MSLN-TTC treatment also promoted the migration of CD103^+^ DCs and the infiltration of CD8^+^ T cells into the tumor microenvironment, an effect that was further enhanced when the treatment was combined with anti-PD-L1 therapy [15]. A similar approach was employed using the alpha-emitting radiopharmaceutical astatine-211-labeled MM4 (^211^At-MM4), which targets poly(ADP-ribose) polymerase 1 (PARP1), to evaluate the combined therapeutic effect of alpha radiation and PD-1 immune checkpoint blockade in a syngeneic mouse model of glioblastoma. Mice received a dose of 36 MBq/kg. The tumors treated with anti-PD-1 alone exhibited infiltration by CD4^+^ and cytotoxic CD8^+^ T cells. In contrast, treatment with ^211^At-MM4 alone resulted in a notable increase in tumor-associated macrophages and CD4^+^ T cells, but this was accompanied by a reduction in CD8^+^ T-cell infiltration. Strikingly, the combination of ^211^At-MM4 with anti-PD-1 therapy resulted in complete tumor regression in 100% of the treated mice [37].

In preclinical studies, treatment with ^90^Y-NM600 demonstrated the significant induction of IFN responses across multiple cancer models, including melanoma (B16 and B78 cell lines) and head and neck cancer (MOC2). This activation was observed at all tested doses—approximately 2.5 Gy and 12 Gy cumulative absorbed doses in the tumor—and was shown to be STING-dependent. These findings suggest that IFN-mediated STING activation is influenced more by temporal dynamics than by the intrinsic susceptibility of tumor cells to DNA damage. Moreover, the combination of ^90^Y-NM600 with immunotherapy resulted in reduced tumor growth and prolonged survival in the murine model of head and neck cancer [39].

On the other hand, hypoxia within the tumor microenvironment adversely affects the proliferation and functional competence of CD8^+^ T cells. Under hypoxic conditions, these cells exhibit the upregulated expression of inhibitory immune checkpoint molecules and increased levels of granzyme B, a response that is modulated by hypoxia-inducible factor 1-alpha (HIF-1α). This altered immune profile is associated with the reduced secretion of critical effector cytokines, including IFN-γ and IL-2, and thereby impairs the antitumor immune response (Figure 2) [124].

The proliferation rate of tumor cells often exceeds the capacity of neovascularization, which results in an insufficient oxygen supply within the tumor mass. To survive under these hypoxic conditions, malignant cells undergo metabolic reprogramming that supports their continued growth and survival, which results in a series of adaptive responses that culminate in enhanced radioresistance [125]. Hypoxia is a hallmark of many solid tumors and often arises from the excessive proliferation of tumor cells, which leads to oxygen depletion in the tumor core. This oxygen limitation stabilizes HIF-1α, a transcription factor that enhances the expression of pyruvate dehydrogenase kinase 1 (PDK1). PDK1 inhibits the conversion of pyruvate to acetyl-CoA and thereby restricts the entry of glucose-derived carbon into the Krebs cycle and promotes lactate production through anaerobic glycolysis [124]. The radiopharmaceutical I-GAZ, a non-glycosidic nitroimidazole-6-O-glucose adduct, was developed as a substrate of glucose transporter 1 (GLUT1) for the diagnosis and treatment of tumors under hypoxic conditions. In breast epithelial cells, ^131^I-GAZ exhibited a 50% higher uptake under hypoxia, which indicated hypoxia-dependent accumulation. However, this uptake was independent of the extracellular glucose levels, which suggested that GLUT1-mediated transport is not the primary mechanism of cellular internalization [126].

## 5. Overall Efficacy of Targeted Radionuclide Therapy Mediated by Long-Term and Non-Targeted Effects

The primary biological challenges associated with achieving optimal efficacy for TRT include the heterogeneous expression of molecular targets, clonal selection phenomena, heterogeneous dose distribution, tumor perfusion, and retention. To enhance the clinical effectiveness of TRT, it is crucial to comprehend the molecular mechanisms underlying the non-targeted effects (NTEs) of TRT. Among the main NTEs produced by ionizing radiation that TRT can trigger are the following: RICEs, RIBEs, low-dose hyper-radiosensitivity (LDHR), abscopal effects (AEs), RIGI, and radiation-induced adaptive responses (RIARs).

Although the preponderance of knowledge concerning the NTEs induced by ionizing radiation emanates from the assessment of radiobiological responses that result from exposure to external sources [127,128], it is reasonable to hypothesize that the molecular mechanisms underlying the NTEs induced by radiation by external beams exhibit parallels with those triggered by TRT, even though it must be demonstrated.

RICEs, one of the NTEs, locally manifested as a heightened cytotoxic response within the irradiated area following non-uniform irradiation. The communication between cells that have received disparate radiation doses (cells that received a higher radiation dose communicate via gap junctions and through soluble factors with those that received a lower radiation dose) generates a more pronounced cytotoxic response in those cells that received low radiation doses, which elicits a global response from all the surrounding cells. Given that heterogeneous dose distribution is a common consequence of variable and dynamic target expression in cancer cells, RICEs induction would likely occur in TRT and be highly favorable, considering the adverse impact of a heterogeneous dose distribution on TRT outcomes [123].

Although some reports highlight the radiobiological benefits of RICEs induced by X-rays and demonstrate that cells exposed to higher doses secrete molecules that increase oxidative stress and induce cell death, RICEs represent a lesser-understood non-targeted effect. Zhang, in 2016, reported that MCF7 cell cultures that were exposed to dose gradients (2–8 Gy) exhibited greater apoptotic cell death, more oxidative stress, and larger equivalent doses than those that received a 5 Gy uniform dose, while Therashima demonstrated, in co-cultures constructed of A549 and SAScells maintaining direct or indirect cell-to cell-contact and irradiated with higher (4 Gy) or lower (0.8 or 3.2 Gy) radiation doses, that the cells that received higher radiation doses sent molecular cohort signals that reduce the damage (oxidative stress and apoptosis) to the cells that received lower doses [129].

Although both RICEs and RIBEs correspond to local NTEs that occur within a 5 mm perimeter, RICEs describe the interactions and effects within an irradiated cell population. At the same time, RIBEs refer to the influence of irradiated cells on nearby non-irradiated cells. As established in the preceding articles, the non-directed effects produced by RICEs are contrary to those made by RIBEs [2].

When RIBEs occur, ROS, superoxide radicals, and lipid peroxidation metabolites are produced in excess and diffuse from irradiated to non-irradiated cells through gap junctions and exosomes. These molecules then cause DNA damage, which is sensed by TP53, TNF-α, and IL-8 and thus triggers the subsequent activation of ATM/ATR/Chk1, which halts the progression of the cell cycle. The cell arrest that results from RIBEs is context-dependent, exhibiting protective or toxic consequences depending on the specific cell type and its respective microenvironment.

RIBEs induction by TRT has been the subject of extensive analysis. Preliminary reports using tritiated thymidine (^3^H-dThd) or 125-Iodine-5-iodo-2′-deoxyuridine (^125^I-UdR) in 3D cocultures and spheroids have indicated that, in the short term, these selectively up-taken toxic nucleosides produce oxidative stress [130] and that, in vivo, RIBEs can be triggered by the deposition of a concentrated amount of energy from Auger and Coster–Kronig electrons [131]. Other experimental strategies that combine gene therapy with TRT in a transfected mosaic spheroid model (TMS) have shown the therapeutic advantage of inducing distant effects, especially with high-LET radionuclides, such as the alpha emitter Astatine-211 [4].

To assess the role of radiation quality in promoting RIBEs that occur due to TRT, Boyd and colleagues utilized glioma (UVW) and bladder carcinoma (EJ138) cells that had undergone genetic modification to alter the expression pattern of the noradrenaline transporter. This modification enabled the regulation of these cells’ capacity to uptake and internalize Meta-iodobenzylguanidine (MIBG) radio-analogs labeled with disparate radionuclides, which differed in their emission spectrum and linear energy transfer (LET). These experiments enabled the determination of the relevance of the dose distribution in the tissue for the induction and comparison of the cytotoxic NTEs produced by a beta particle emitter, ^131^I-MIBG (LET = 0.2–2 keV/µm and mean range of 0); an electron auger emitter, ^123^I-MIBG (LET = 4–26 keV/µm and mean range of <5 µm); and an alpha particle emitter, ^211^At-MABG (LET = 80 keV/µm and a mean range of 0.04–0.1 mm). Boyd and colleagues figured out the survival fraction by conducting clonogenic assays of the cells that were treated with conditioned culture media recovered from culture flasks of cells that had been irradiated with gamma rays (0–9 Gy) from a source of ^60^Co, ^131^I-MIBG, ^123^I-MIBG (0–11 MBq/mL), or ^211^At-MABG (0–45 kBq/mL). These experiments demonstrated that the specificity and internalization of the radiopharmaceutical must be considered to promote RIBEs, as the non-internalized radiopharmaceutical fraction proved incapable of triggering RIBEs under the analyzed experimental conditions. In the context of TRT, the selection of radionuclides assumes significance since, in contrast to the behavior observed with Auger and alpha emitters (^123^I-MIBG and ^211^At-MABG1), the low-LET beta emitter (^131^I-MIBG) induced the greater NTEs in both examined cell lines (80% cell death in UVW/NAT cells and 72% in EJ138/NAT cells), without showing RIBEs on the cell killing saturation at increasing radiopharmaceutical activity concentrations [132].

In a study conducted by Grzmil, the responses induced by the radiolabeled minigastrin analog ^177^Lu-PP-F11N were examined and compared with those caused by external radiation gamma beam exposure. The effects on proliferation were analogous. Proteomics, however, exhibited marked distinctions. The two therapeutic interventions induced p53-mediated pathways associated with DNA damage detection, as well as cell-cycle regulation and RNA processing, metabolism, cell transport, morphology, and adhesion. It has been demonstrated that TRT-induced signaling occurs through multiple receptors, including the transforming growth factor beta receptor (TGFβR), EGFR, hepatocyte growth factor receptor (HGFR), mechanistic target of rapamycin receptor (mTOR), and Rho homolog (Rho), integrin, and estrogen receptors.

In contrast, external beam radiotherapy (EBRT)-induced signaling has been found to involve the RAS, ATM, PYK, and MAPK pathways. Divergent responses were also observed in integrin and c-Jun expression, EGFR phosphorylation, and extracellular signal-regulated kinase 1/2 (ERK1/2) activation; these were increased by TRT and decreased in response to EBRT. The disparities in signaling elicited by these two types of radiotherapy may be associated with the nature and magnitude of the induced DNA damage [9]. These disparities are influenced by the dose distribution within subcellular compartments and by the proportion of irradiated cells in the tumor mass [4,130,131].

LDHR is considered an NTE, being characterized by an excessive sensitivity to radiation doses that results from prior exposure to radiation doses below 1 Gy, which is reported for both low- and high-LET radiation. Experiments performed by Ye et al. [133], consisting of the exposure of normal lung fibroblasts (MRC-5) to high-LET carbon ions in the presence or absence of gap junction inhibitors, showed that direct radiation interactions induce LDHR at doses <0.2 Gy and cannot be considered as RIBEs. Additionally, they reported that low-dose-induced biological effects (clonogenicity, DNA damage, and micronuclei formation) were suppressed under gap junction inhibition [133].

The etiology of LDHR remains ambiguous; however, it appears to be a consequence of the accumulation of a substantial number of DSBs, which results in the hyperactivation of the DNA repair machinery, including the phosphorylation of ATM, the master sensor of DNA damage. Despite the lack of knowledge regarding the mechanism that results in the loss of ATM’s sensor ability, it is conceivable that this occurrence may happen during the LDHR process [134].

After a broad-scope overview of the state of the art, we found no studies mentioning the induction of LDHR by TRT; however, in the contemporary context of oncology, multimodality treatments have demonstrated superior efficacy compared to single therapies. LDRT, defined as the administration of doses below the direct cytotoxic threshold, has emerged as a promising strategy for modulating the TME and enhancing the antitumor immune response. LDRT exhibits not only excellent tolerability and a low cost but also minimal toxicity. Furthermore, LDRT is compatible with other therapeutic modalities, such as surgery, chemotherapy, and immunotherapy. Preclinical investigations in animal models of high-grade gliomas (HGGs) have demonstrated that implementing hyper-fractionation schemes (≤0.5 Gy twice a week) during the early phases of tumor development significantly enhances the efficacy of radiotherapy and prolongs survival.

From a mechanistic perspective, LDRT with doses ranging from 0.5 to 2 Gy appears to elicit a multifaceted response (TME remodeling, neo-angiogenesis, T-cell infiltration, activation of pro-inflammatory signaling, and enhancement of the immune response) that surely involves LDHR and that effectively delays tumor progression and metastasis [8]. Recently, our group has demonstrated that the in vitro treatment of prostate cancer cells (LNCaP) with gamma radiation doses (<0.5 Gy) before TRT with ^177^Lu-iPSMA (0.48 Bq/cell) significantly reduces the cell survival by inducing late apoptosis [unpublished results]. These findings highlight the immunomodulatory and therapeutic promise of the LDHR effect induced by LDRT or TRT as a complementary strategy in oncologic treatment.

To capitalize on the induction of the LDHR phenomenon as a component of a clinical strategy to enhance TRT, it is crucial to inhibit the activation of mechanisms that foster insensitivity, as this can lead to adaptive responses by increasing radioresistance [135].

A strategy to enhance TRT would entail inducing hypersensitivity phenomena without concomitant genomic insensitivity and adaptation, which could be achieved by circumventing the activation of radioresistance mechanisms.

The analysis of the secretome induced by TRT revealed that neuroblastoma cells (SK-N-BE) treated with ^131^I-MIBG produced ROS, interleukin-6 (IL-6), and tumor necrosis factor-α (TNF-α). Prostate cancer cells (LNCaP and PC3) treated with ^223^RaCl_2_ exhibited increased calreticulin, HMGB1, and extracellular adenosine triphosphate (ATP) levels. While interleukin-1β (IFβ), chemokine (C-X-C motif) ligand 10 (CXCL10), and IL-8 were detected in neuroendocrine tumors (NCL H727) treated with ^177^Lu-DOTA-TATE, other studies have reported the presence of FasL, TRAIL, and IL-1β in in vivo models of non-Hodgkin’s lymphoma treated with ^90^Y-ibritumomab tiuxetan. Furthermore, analysis of the murine models of breast cancer treated with ^131^I-labeled albumin nanoparticles has demonstrated the presence of IL-6 and MMP-9. Similarly, in patients with hepatocarcinoma treated with ^90^Y-labeled microspheres, the synthesis of VEGF, MMP-2, and IL-8 was induced. In general, the soluble factors produced and secreted after TRT can be categorized as follows: (i) proinflammatory cytokines (TNF-α, IL-1β, IL-6, and IL-8, which have been shown to induce inflammation and activate immune responses), (ii) chemokines (CXCL10, CCL2, and CXCR6, which have been identified as promoters of T-lymphocyte and macrophage infiltration), (iii) immunogens (DAMPs, HMGB1, ATP, calreticulin, extracellular), (iv) immune system reactivators (DNA fragments via the cGAS-STING pathway), (v) reactive species (ROS and RNS, which have been shown to initiate NTEs, particularly RICEs and RIBEs), (vi) interferon (IFN-β, which has been identified as a key modulator of immune responses), (vii) apoptotic factors (FasL and TRAIL), and (viii) angiogenic and remodeling factors (VEGF and MMPs) [136].

The soluble factors that comprise the secretome of irradiated tumor cells, in addition to local effects (RICEs and RIBEs), can alter cell behavior through AEs, triggering antitumor mechanisms at a distance from the irradiated area by releasing short- and long-distance messengers. These messengers are responsible for conveying stress signals to distant sites through inflammatory and immune signaling networks. The biological outcomes promoted by AEs are diverse because they are contingent upon biochemical cell conditions and the composition of the tumor microenvironment. It is worth noting that the AEs are triggered upon exposure to radiation doses that are higher (2–40 Gy) than those that elicit RIBEs. As multiple authors have asserted, the effectiveness of AEs is contingent upon the immune system and its antitumor actions, which are associated with cell stress and utilize various signaling pathways. For instance, it has been observed that the action of HSP70 and box-1 is mandatory for dendritic cell activation. Additionally, GRP78 has been demonstrated to stimulate cell proliferation and migration by interacting with Crypto-1 and activating the PI3K/Akt pathway. Concurrently, cellular stress, whether induced by GRP78 or alternative mechanisms, has been observed to promote the epigenetic transcriptional deregulation of oncogenes (c-Myc) and histones through NF-κB, STAT3, and SMAD [137].

It is essential to consider that pathological conditions such as cancer, as well as their treatment (chemo and radiotherapy), act by inducing cellular stress. The initial step of NTEs is cellular stress induced by radiation, and its effects on specific cell organelles (mitochondria, endoplasmic reticulum, and stress granules) may act directly and indirectly to sustain NTEs.

Mitochondria are among the most significant cell organelles, second only to the cell nucleus. This organelle plays a pivotal role in regulating metabolism, bioelectrical activity, energy balance, epigenetics, and innate immunity [16]. Ionizing radiation has been shown to cause damage to mitochondrial DNA (mtDNA), which results in an energetic imbalance, the formation of ROS, cellular stress, and the initiation of cell apoptosis. The persistent damage to mtDNA, in conjunction with the other radiation-induced mitochondrial responses, has been shown to contribute to RIGI and RIARs [138]. Several mitochondrial mechanisms have been identified as being involved in RIGI and RIARs. These include mtDNA radiosensitivity resulting from the absence of histones and efficient repair, as well as the promotion of nuclear DNA damage, including epigenetic modifications mediated by mitochondrial changes. Additionally, biogenesis and the regulation of inflammation and immune responses are facilitated through the activation of cGAS-STING [139].

The endoplasmic reticulum plays a pivotal role in several critical processes, including calcium storage, protein synthesis, and lipid metabolism. The subsequent accumulation of mutated and misfolded proteins, resulting from cell irradiation, has been demonstrated to trigger the activation of the unfolded protein response (UPR) signaling pathway. This activation, in turn, leads to the secretion of heat shock proteins (Hsp60, Hsp90, or GRP78) in extracellular vesicles. The relocation of GRP78 to the cell surface (CS-GRP78) has been demonstrated to promote an adaptive response, leading to resistance to treatment and the expression of stem-like, radioresistant phenotypes, which in turn influence long-term radiation responses [140].

Stress granules (SGs) are organelles that form as a transient response to cellular stress. They play a critical role in regulating protein synthesis by temporarily sequestering mRNA and proteins. Analysis of the composition of SGs reveals that the presence of mRNA and proteins within them plays a regulatory role in key signaling pathways that control cell survival, repair, and resistance. SG formation induced by radiation facilitates cell metabolic adaptation and RIARs by inhibiting the synthesis of pro-apoptotic proteins, favoring DNA damage repair, and reducing ROS formation [141].

Clastogenic factors are mutagenic agents (tumor growth factors, lipid peroxidation products, and atypical nucleotides) that can be detected in the plasma of stressed organisms for extended periods. Clastogenic factors provoke a large number of cellular and molecular changes (chromosomal breaking, chromosomal aberrations, chromosomal misrepair, cell killing, and transformation) as part of the AEs that can lead to RIARs [142].

The capacity of a cell to withstand subsequent exposure to a higher absorbed dose of radiation after previous exposure to low doses of radiation is referred to as its RIARs. The mechanisms underlying RIARs include a decrease in TP53, the production of ROS, the activation of redox enzymes, and the promotion of the BER pathway. A multitude of soluble factors are released during RIARs, with the predominant subset comprising those that modify nuclear proteins involved in maintaining the chromatin structure and DNA replication [143].

The massive production and accumulation of radiation-induced mutations is a consequence of the loss of DNA replication fidelity and inefficient repair machinery. Together, these factors induce the complex, inter-coordinated mechanisms underlying RIARs: genetic instability, genetic susceptibility, hormesis, immune response activation, and stem cell repopulation. Some cellular adaptations that occur during RIARs modify the epithelial–mesenchymal transition program, altering the immune and inflammatory responses. These adaptations encompass gene expressions, microRNA synthesis, altered lipid metabolism, and signaling via the AKT/mTOR pathway [144,145].

Clastogenic factors are mutagenic agents (e.g., cytokines, tumor growth factors, lipid peroxidation products, and atypical nucleotides) that appear approximately 20 h post-irradiation and can be detected for extended periods (years) in the plasma of stressed organisms. These factors provoke numerous cellular and molecular changes, including chromosomal breaks, chromosomal aberrations, chromosomal misrepair, cell death, and cell transformation, which lead to RIARs by inducing long-term inflammatory responses [146].

Both RIGI and RIARs are long-lasting NTEs that involve long-distance stochastic phenomena like RIBEs. In both cases, the release of soluble factors through extracellular vesicles (e.g., exosomes, microvesicles) is indispensable [128].

The composition and release rate of TRT-induced extracellular vesicles vary depending on the radionuclide. This variation could produce contradictions in TRT-induced RIARs (treatment resistance or immune checkpoint uncoupling). For this reason, recurrence and treatment resistance observed after TRT could be a consequence of genetic instability and hormesis induced by sublethal damage to non-target tissue caused by TRT. It is worth mentioning that both adaptive mechanisms are triggered by low-dose radiation exposure [135].

## 6. Conclusions and Future Directions

Optimizing radiotherapy using radiopharmaceuticals presents numerous challenges, with accurate dosimetry being one of the most frequently addressed aspects in improving these therapies. Although patient-specific dosimetry is not yet a standardized practice, its implementation is crucial due to the variability in the distribution of the absorbed dose, particularly in critical organs such as the kidneys. Furthermore, developing personalized treatment plans that take into account individual patient characteristics and tumor biology is a complex process that requires advanced imaging techniques and sophisticated modeling tools, which have been rapidly evolving, to accurately predict treatment outcomes.

This review underlined the achievements in treating patients with prostate cancer and advanced neuroendocrine tumors using radiopharmaceuticals under a theranostic modality. These results represent only the beginning of the potential applications of personalized TRT as an approach to various cancer treatments. However, expanding the use of TRT to treat numerous patients with multiple types of neoplasms requires a significant multidisciplinary effort to address and overcome the substantial challenges related to the molecular pathways that affect cancer-cell toxicity and death.

TRT has demonstrated improvements in survival rates, particularly for patients with prostate cancer. However, 30–40% of patients do not respond to this treatment [64,65,66], which highlights the need for a more comprehensive understanding of tumor microenvironment dynamics, the long-term and non-targeted effects of TRT, and the molecular pathways involved in cell death and survival, as well as the necessity of implementing improved combined therapies. Historically, many radiopharmaceuticals have shown limited survival benefits. For example, bone radiopharmaceuticals are typically restricted to palliative care in cases of bone metastases [147]. This fact may be due in part to the lack of studies linking the effects of radiopharmaceuticals to molecular mechanisms within the tumor microenvironment.

Radiation resistance arises when tumor cells activate survival pathways in response to treatment and makes it challenging to eliminate malignant cells effectively. Additionally, the tumor microenvironment presents conditions such as hypoxia and the presence of immune cells, which reduce the efficacy of TRT. Furthermore, cancer cells can evade cell death pathways, such as apoptosis, which exacerbates resistance to treatment. Therefore, advancing research aimed at designing radionuclides linked to targeted molecular agents that modulate cytoprotective and cytotoxic pathways is imperative to improving outcomes.

Despite the fact that guidelines concerning the administration of TRT in clinics focus on the application of radiopharmaceuticals in conjunction with immunotherapy [13], these approaches may increase treatment toxicity. The use of combination therapies adds complexity, as balancing efficacy and toxicity involves the use of dosimetric and radiobiological models to ensure that normal organs do not exceed their tolerance levels.

Several clinical and preclinical studies on radiopharmaceuticals that combine TRT with immunotherapy, such as the use of iPD-L1, have demonstrated an enhanced immune response against tumors [30] (Table 1). However, further research is also needed to investigate the effects of TRT on hypoxia, glycolysis, and the metabolic reprogramming mechanisms of cancer cells within the tumor microenvironment. Such studies are crucial for the development of a broader range of combined therapies that can improve patient outcomes.

As described above, radiotherapy outcomes are enhanced by the induction of non-targeted effects, RICEs, RIBEs, LDHR, AEs, RIGI, and RIARs. However, it is essential to note that TRT also faces numerous challenges due to the genetic heterogeneity and mutation complexity within tumors. Tumors often consist of diverse cell populations with different genetic profiles, which leads to varied responses to treatment within the same tumor [148,149]. This genetic instability and heterogeneity complicate the success of TRT. Distinguishing driver mutations (those that contribute to cancer progression) from passenger mutations (those that do not affect cancer growth) is a challenging yet crucial task for TRT. Genetic mutations can lead to both primary radioresistance (where the cancer does not respond to treatment from the start) and acquired radioresistance (where the cancer initially responds but later becomes resistant). Moreover, subclonal populations of cancer cells may endure initial treatments and lead to relapse, which makes it essential to identify and target these resistant cells. Therefore, future directions in the development of radiopharmaceuticals must also address the need for a better understanding of key mutations in tumors to develop validated target-specific molecules that carry therapeutic radionuclides that contribute to ensuring the efficacy of TRT.

## 7. Summarizing Limitations and Challenges of Targeted Radionuclide Therapy

Accurate dosimetry: One of the primary challenges in optimizing TRT is achieving accurate dosimetry, which is crucial for improving therapies, as the variability in the radiation absorbed dose in critical organs, such as the kidneys, can significantly impact treatment outcomes.

Personalized treatment plans: Developing personalized treatment plans that consider individual patient characteristics and tumor biology is a complex process. This process requires advanced imaging techniques and sophisticated modeling tools, which are rapidly evolving to accurately predict treatment outcomes.

Tumor microenvironment: The tumor microenvironment presents conditions such as hypoxia and the presence of immune cells, which reduce the efficacy of TRT. Understanding the dynamics of the tumor microenvironment and its impact on cancer-cell behavior is crucial for enhancing treatment outcomes.

Radiation resistance: Tumor cells can activate survival pathways in response to treatment, making it challenging to effectively eliminate malignant cells. Additionally, cancer cells can evade cell death pathways, such as apoptosis, exacerbating resistance to treatment.

Genetic heterogeneity and mutation complexity: Tumors often consist of diverse cell populations with distinct genetic profiles, which results in varied responses to treatment within the same tumor. This genetic instability and heterogeneity complicate the success of TRT. Distinguishing driver mutations from passenger mutations is crucial for ensuring the efficacy of TRT.

Non-targeted effects: TRT induces non-targeted effects, including RICEs, RIBEs, LDHR, AEs, RIGI, and RIARs. These effects need to be better understood to enhance the outcomes of TRT. For this purpose, the development of novel pre-clinical cancer models that can distinguish between the known physical radiation effects and the secondary activation of signaling pathways triggered by radiopharmaceuticals is primarily essential.

Combination therapies: While combining TRT with immunotherapy shows promise, it may potentially increase the treatment’s toxicity. Balancing efficacy and toxicity involves the use of dosimetric and radiobiological models to ensure that normal organs do not exceed their tolerance levels.

These limitations underscore the need for a multidisciplinary approach to address and overcome the substantial challenges related to molecular pathways that affect cancer-cell toxicity and death, as well as the necessity of implementing improved combined therapies.

## Figures and Tables

**Table 1:** Effects on immune checkpoints (ICs) of targeted radiopharmaceuticals.

Radiopharmaceutical	Target	IC Involved	Tumor Model	Results	References
^177^Lu-LLP2A	Very late antigen-4	PD-1/PD-L1, CTLA-4	murine B16-F10 melanoma	Significant levels of apoptosis increases survival	[30]
^177^Lu-EB-cRGDfK	integrin α_v_β_3_	PD-L1	murine MC38 colon adenocarcinoma	Increases PD-L1 expression on T cells, reduces tumor volume, and increases overall survival	[9]
^131^I-ICF01012	melanin	PD-1/PD-L1, CTLA-4	murine B16-F10 melanoma	Increases survival from 24 to 41 days	[31]
^213^Bi-h8C3	melanin	PD-1	murine B16-F10 melanoma	Reduces tumor growth and increases survival without decreasing animal body weight	[32]
^177^Lu-h8C3	melanin	PD-1	Cloudman S91 murine melanoma	Reduces tumor growth and increases survival	[33]
^225^Ac -h8C3	melanin	PD-1	Cloudman S91 murine melanoma	No therapeutic efficacy	[33]
^227^Th-TTC	mesothelin	PD-L1	The murine cell line MC38 transfected with the human gene encoding for MSLN (hMSLN)	Increases the CD8 T-cell infiltration and the number of tumor-free surviving animals	[15]
^177^Lu-Lumi804-αCD11	CD11b+ cells	PD-1 CTLA-4	GL261 glioma	Enhances the efficiency of the dual IC without altering the composition of immune cells within the TME	[34]
^177^Lu-anti-PD-L1	PD-L1	PD-L1	MC38 murine colon adenocarcinoma	Increases the infiltration of CD4+ and CD8^+^ T-cells	[35]
^177^Lu-DNP-DOTA-BSA	No target	CTLA-4 PD-L1	E0771 murine triple-negative breast cancer	Increases necrotic tissue within the tumor and decreases levels of F4/80+ macrophages	[36]
^211^At-MM4	PARP-1	PD-1	GL261 glioblastoma	Decreases tumor volume, disease-free mice were 100%.	[37]
^225^Ac-PSMA	PSMA	PD-1	RM1-PGLS prostate cancer	Enhances the efficiency of PD-1	[38]
^90^Y-NM600	lipid rafts	PD-1 CTLA-4	MOC2 head and neck squamous cell carcinoma	Reduces tumor growth and increases survival	[39]
^90^Y-NM600	lipid rafts	PD-1 CTLA-4	B78 melanoma, B16 melanoma, 4T1 breast cancer NXS2 neuroblastoma	Low absorbed doses (2–5 Gy) activate the production of cytokines in TME, promoting tumor infiltration	[40]
^212^Pb-VMT01	melanocortin receptor	PD-1 CTLA-4	B16F10 melanoma YUMM1.7 melanoma	Enhances infiltration of CD3^+^, CD4^+^ and CD8^+^ lymphocytes 43% of the mice showed complete tumor response	[41]
^177^Lu-DOTA-folate	Folate receptor	CTLA-4	NF9006 breast cancer	Reduces tumor growth and increases survival	[42]
^90^Y-GZP	GranzymeB	PD-1 CTLA-4	MC38 colon adenocarcinoma CT26 colon carcinoma	Promotes a dose-dependent response and increases survival	[43]
^131^I-anti-PD-L1	PD-L1	PD-L1	MC38 colon adenocarcinoma CT26 colon carcinoma	Delays significant tumor growth and prolongs survival	[44]
^90^Y-NM600	lipid rafts	PD-1 CTLA-4	TRAMP-C1 prostate cancer, MycCAP Prostate cancer	Ineffective in the prostate cancer models studied	[45]
^177^Lu-DOTA-EB-cRGDfK	integrin α_v_β_3_	PD-L1	MC38 colon adenocarcinoma CT26 colon carcinoma	Inhibits tumor growth and protects against tumor recurrence	[46]
^177^Lu-iPD-L1	PD-L1	PD-1/ PD-L1	4T1 tumors Murine triple-negative breast cancer	Substantial increase in activated macrophages, IL-10, TGF beta, and PD-L1 expression levels	[47]
^177^Lu-FAP-2286	FAP	PD-1	MCA205 mouse FAP-expressing tumors	Modulates the TME and increases CD8^+^ T-cell infiltration, significantly inhibiting tumor growth and improving survival	[8]
^177^Lu-LNC1004	FAP	PD-L1	MC38/NIH3T3-FAP and CT26/NIH3T3-FAP tumor xenografts	suppression of malignant progression and increasing cell-to-cell communication, CD8^+^ T-cell activation, and expansion	[48]
^177^Lu-DOTA-girentuximab	carbonic anhydrase IX (CAIX)	PD-1 CTLA-4	Renca-CAIX or CT26-CAIX renal cell carcinoma	T-cell infiltration and modulated immune signaling pathways in the TME with complete tumor remission.	[49]
^177^Lu-DOTA-2P(FAPI)_2_	FAP	PD-L1	CT26-FAP colorectal tumor	Tumor suppression, infiltrating CD8^+^ T-cells, and 100% tumor rejection after tumor cell re-inoculation	[50]
^177^Lu-AB-3PRGD_2_	integrin α_v_β_3_	PD-L1	MC38 colon adenocarcinoma, B16-F10 melanoma	Tumor suppression, infiltrating CD8^+^ T-cells	[51]

**Table 2:** Clinical outcomes of ^177^Lu radiopharmaceuticals used in patients with neuroendocrine tumors (NETs) and metastatic castration-resistant prostate cancer (mCRPC).

Radiopharmaceutical (Cancer Type)	Median OS (Months)	PFS (Months)	Response Rates	References
^177^Lu-DOTA-TATE (NETs)	>48	25.6	Symptomatic improvement: 71.4% Partial response: 66.7%	[60,61]
^177^Lu-DOTA-TOC (NETs)	>44.2	34.7	Objective response: 33.9% Disease control: 66.1%	[62,63]
^177^Lu-DOTA-iPSMA (mCRPC)	21.7	10.6	PSA decline: 73% Pain reduction: 88%	[63]
^177^Lu-PSMA-617 (mCRPC)	15.3 (34 months if applied before chemotherapy and combined with enzalutamide)	8.7 (11.6 months if applied before chemotherapy)	PSA decline ≥50%: Disease control: 62%	[64,65,66]
^177^Lu-PSMA I&T (mCRPC)	17.1	7.4	PSA decline of ≥50%	[67]

**Table 3:** Evaluation of apoptosis induced by using therapeutic radiopharmaceuticals as a tool to monitor the efficacy of cancer treatment.

Radiopharmaceutical	Cancer Type	Therapeutic Target	Biomarker	Activity/Dose	Mechanism of Apoptosis Induction	References
^177^Lu-DOTA-TATE + onalespib	Neuroendocrine tumors	Somatostatin receptors (SSTR2)	Increase activity in caspase 3/7, γH2AX, p53,p21 y BAX. Reduction EGFR	5 kBq +	Binding to SSTR2 receptors, internalization, and release of radiation into the cell → DNA damage (level of DNA double-strand breaks) → apoptosis	[98,99]
^177^Lu-DOTA-Miltuximab	Prostate cancer	Glypican-1 (GPC-1)	Cleaved caspase 3	6 MBq +	Miltuximab binds specifically to Glypican-1, internalization and release of radiation into the cell → DNA damage (level of DNA double-strand breaks)→, activation of caspases 3 and 9 (intrinsic pathway), and cell apoptosis.	[100]
^177^Lu-PSMA-617	Metastatic prostate cancer	Prostate-specific membrane antigen (PSMA)	Increase activity in γ-H2AX+53BP1	6.0 GBq (01 Gy to blood) +++	DNA damage caused by beta emission generates free radicals, activating DNA damage pathways that lead to the activation of caspases (intrinsic pathway) and cell apoptosis.	[78]
^177^Lu-trastuzumab	Metastatic breast cancer	HER2-positive tumors	Activate caspase 3, and PARP interferes with DNA-PK expression	13.8 MBq ++	HER2-specific binding induced cell death (DNA double-strand breaks), activation of p53, ATM, ATR, cytochrome c release, activation of caspases 9 and 3 (intrinsic pathway), and cell apoptosis.	[101]
^90^Y-Ibritumomab	Non-Hodgkin’s lymphoma	CD20 antigen on B-cells	Arrest of cells in the G(2)/M phase of the cell cycle increases activity in caspase	0.3 to 0.4 Gy ++	Binding to CD20 → internalization → radiation damage (DNA double-strand breaks) → apoptosis via caspase activation and mitochondrial damage.	[102]
^223^RaCl_2_	Castration-resistant prostate cancer (CRPC) with bone metastases	selectively binds to hydroxyapatite in bone	Increase of γ-H2AX	300 kBq/kg ++	High LET → irreversible DNA damage (DNA double-strand breaks) → apoptosis	[103]
^225^Ac-E4G1	Tumor neovasculature in prostate cancer models.	Antibody E4G10 specifically binds the form of VE-cadherin	Increase in cleaved caspase-3	1.85 KBq ++	High LET → irreversible DNA damage (DNA double-strand breaks) → apoptosis	[104]
^131^I-MIBG	Neuroendocrine tumors, such as neuroblastoma and pheochromocytoma/paraganglioma.	Cells with norepinephrine uptake	Increase in cleaved caspase-3 and PARP, Cell-cycle arrest in G2/M	37 MBq ++	Accumulates in synaptic vesicles → intracellular damage by β- → activation of the mitochondrial apoptosis pathway.	[105]
^213^Bi-DOTA-TOC	Neuroendocrine tumors	Somatostatin receptors (SSTR2)	release of apoptosis-specific mono-nucleosomes and oligonucleosomes	37 KBq +	Binding to SSTR2 receptors, internalization, High LET → lethal DNA damage (DNA double-strand breaks) → immediate apoptosis in tumor cells	[106]
^225^Ac-PSMA-RGD	Metastatic prostate cancer and tumor angiogenesis	PSMA, integrins	Increase in caspase-3/7	2–4 Gy +	Binding to PSMA and integrins, internalization, High LET → lethal DNA damage (DNA double-strand breaks) → immediate apoptosis in tumor cells	[22]

+ in vitro; ++ tumor mouse; +++ blood samples from patients.

## Data Availability

Data is contained within the article.

## Abbreviations

The following abbreviations are used in this manuscript:

AEs	Abscopal effects
APCs	Antigen-presenting cells
ATM	Ataxia telangiectasia mutated
ATR	Rad3-related kinases
BAK	BCL2 homologous antagonist killer
BAX	BCL2-associated X
BER	Base excision repair
BRCA2	Breast Cancer gene 2
CAFs	Cancer-associated fibroblasts
cGAS	Cyclic GMP–AMP synthase
CTLA-4	Cytotoxic T-lymphocyte antigen 4
DAMPs	Damage-associated molecular patterns
DCs	Dendritic cells
DDR	DNA damage response
DNA-PK	DNA-dependent protein kinase complex
DOTA	1,4,7,10-tetraazacyclododecane-1,4,7,10-tetraacetic acid
DSBs	Double-strand breaks
EGFR	Epidermal growth factor receptor
FAP	Fibroblast activation protein
FEN1	Flap endonuclease 1
GLUT1	Glucose transporter 1
HIF-1α	Hypoxia-inducible factor 1-alpha
HMGB1	High-mobility group box 1 protein
HR	Homologous recombination
ICs	Immune checkpoints
iPD-L1	Programmed death ligand 1 inhibitor
LDHR	Low-dose hyper-radiosensitivity
LDRT	Low-dose radiotherapy
MAPK	Mitogen-activated protein kinasa
mCRPC	Metastatic castration-resistance prostate cancer
MHC	Major histocompatibility complex
MIBG	Meta-iodobenzylguanidine
MMR	Non-targeted effects
NETs	Neuroendocrine tumors
NF-κB	Nuclear Factor kappa B
NHEJ	Non-homologous end joining
NK	Natural killer cells
NTEs	Non-targeted effects
OGG1	8-oxoguanina
PD-1	Programmed cell death protein 1
PDK1	Pyruvate dehydrogenase kinase 1
PD-L1	Programmed death ligand 1
PCNA	Proliferating cell nuclear antigen
PSMA	Prostate-specific membrane antigen
RIARs	Radiation-induced adaptive responses
RIBEs	Radiation-induced bystander effects
RICEs	Radiation-induced cohort effects
RIF	Radiation-induced foci
RIGI	Radiation-induced genomic instability
RNS	Reactive nitrogen species
ROS	Reactive oxygen species
SSBs	Single-strand breaks
SSTR	Somatostatin receptors
STING	Stimulator of interferon genes
TAMs	Tumor-associated macrophages
TATE	Tyr^3^-octreotate
TGF-β	Transforming Growth Factor-beta
TOC	Tyr^3^-octreotide
TP53	Tumor Protein 53
TRT	Targeted radionuclide therapy
TTAMs	Tumor-associated macrophages
VLA-4	Very late antigen-4
XLF	XRCC4-like factor
XRCC1	X-ray repair cross-complementing protein 1

## References

[1] Gow M., Seymour C., Boyd M., Mairs R., Prestiwch W., Mothersill C. (2014). Dose Calculations for [131I] Meta-Iodobenzylguanidine-Induced Bystander Effects. Dose-Response.

[2] Terashima S., Tatemura R., Saito W., Hosokawa Y. (2025). Evaluation of the influence of radiation-induced cohort effect in cell populations receiving different doses. Int. J. Radiat. Biol..

[3] Bryant J., Shields L., Hynes C., Howe O., McCleanc B., Lynga F. (2019). DNA damage and cytokine production in non-target irradiated lymphocytes. Radiat. Res..

[4] Boyd M., Mairs R.J., Keith W.N., Ross S.C., Welsh P., Akabani G., Owens J., Vaidyanathan G., Carruthers R., Dorrens J. (2004). An efficient targeted radiotherapy/gene therapy strategy utilising human telomerase promoters and radioastatine and harnessing radiation-mediated bystander effects. J. Gene Med. A Cross-Discip. J. Res. Sci. Gene Transf. Its Clin. Appl..

[5] Fullerton N.E., Mairs R.J., Kirk D., Keith W.N., Carruthers R., McCluskey A.G., Brown M., Wilson L., Boyd M. (2005). Application of targeted radiotherapy/gene therapy to bladder cancer cell lines. Eur. Urol..

[6] Cai Z., Liu R., Chan C., Lu Y., Winnik M.A., Cescon D.W., Reilly R.M. (2022). 90Y-labeled gold nanoparticle depot (NPD) combined with anti-PD-L1 antibodies strongly inhibits the growth of 4T1 tumors in immunocompetent mice and induces an abscopal effect on a distant non-irradiated tumor. Mol. Pharm..

[7] Nabrinsky E., Macklis J., Bitran J. (2022). A review of the abscopal effect in the era of immunotherapy. Cureus.

[8] Zboralski D., Osterkamp F., Christensen E., Bredenbeck A., Schumann A., Hoehne A., Schneider E., Paschke M., Ungewiss J., Haase C. (2023). Fibroblast activation protein targeted radiotherapy induces an immunogenic tumor microenvironment and enhances the efficacy of PD-1 immune checkpoint inhibition. Eur. J. Nucl. Med. Mol. Imaging.

[9] Chen H., Zhao L., Fu K., Lin Q., Wen X., Jacobson O., Sun L., Wu H., Zhang X., Guo Z. (2019). Integrin αvβ3-targeted radionuclide therapy combined with immune checkpoint blockade immunotherapy synergistically enhances anti-tumor efficacy. Theranostics.

[10] Bellavia M.C., Patel R.B., Anderson C.J. (2022). Combined Targeted Radiopharmaceutical Therapy and Immune Checkpoint Blockade: From Preclinical Advances to the Clinic. J. Nucl. Med..

[11] Ferdinandus J., Fendler W.P., Lueckerath K., Berliner C., Kurzidem S., Hadaschik E., Klode J., Zimmer L., Livingstone E., Schadendorf D. (2022). Response to Combined Peptide Receptor Radionuclide Therapy and Checkpoint Immunotherapy with Ipilimumab Plus Nivolumab in Metastatic Merkel Cell Carcinoma. J. Nucl. Med..

[12] Kim C., Liu S.V., Subramaniam D.S., Torres T., Loda M., Esposito G., Giaccone G. (2020). Phase I study of the (177)Lu-DOTA(0)-Tyr(3)-Octreotate (lutathera) in combination with nivolumab in patients with neuroendocrine tumors of the lung. J. Immunother. Cancer..

[13] Aggarwal R., Starzinski S., de Kouchkovsky I., Koshkin V., Bose R., Chou J., Desai A., Kwon D., Kaushal S., Trihy L. (2023). Single-dose (177)Lu-PSMA-617 followed by maintenance pembrolizumab in patients with metastatic castration-resistant prostate cancer: An open-label, dose-expansion, phase 1 trial. Lancet Oncol..

[14] Pouget J.-P., Chan T.A., Galluzzi L., Constanzo J. (2023). Radiopharmaceuticals as combinatorial partners for immune checkpoint inhibitors. Trends Cancer.

[15] Lejeune P., Cruciani V., Berg-Larsen A., Schlicker A., Mobergslien A., Bartnitzky L., Berndt S., Zitzmann-Kolbe S., Kamfenkel C., Stargard S. (2021). Immunostimulatory effects of targeted thorium-227 conjugates as single agent and in combination with anti-PD-L1 therapy. J. Immunother. Cancer.

[16] Zhang S., Wang X., Gao X., Chen X., Li L., Li G., Liu C., Miao Y., Wang R., Hu K. (2025). Radiopharmaceuticals and their applications in medicine. Signal Transduct. Target. Ther..

[17] Ailawadhi S., Pafundi D., Peterson J. (2025). Advances and future directions in radiopharmaceutical delivery for cancer treatment. Expert Rev. Anticancer Ther..

[18] Lawal I.O., Abubakar S.O., Ndlovu H., Mokoala K.M.G., More S.S., Sathekge M.M. (2024). Advances in Radioligand Theranostics in Oncology. Mol. Diagn. Ther..

[19] Barca C., Griessinger C.M., Faust A., Depke D., Essler M., Windhorst A.D., Devoogdt N., Brindle K.M., Schäfers M., Zinnhardt B. (2021). Expanding Theranostic Radiopharmaceuticals for Tumor Diagnosis and Therapy. Pharmaceuticals.

[20] Sharma R., Suman S.K., Mukherjee A. (2022). Antibody-based radiopharmaceuticals as theranostic agents: An overview. Curr. Med. Chem..

[21] Zuo D., Wang H., Yu B., Li Q., Gan L., Chen W. (2024). Astatine-211 and actinium-225: Two promising nuclides in targeted alpha therapy. Acta Biochim. Biophys. Sin..

[22] Ocampo-García B., Cruz-Nova P., Jiménez-Mancilla N., Luna-Gutiérrez M., Oros-Pantoja R., Lara-Almazán N., Pérez-Velasco D., Santos-Cuevas C., Ferro-Flores G. (2023). (225)Ac-iPSMA-RGD for Alpha-Therapy Dual Targeting of Stromal/Tumor Cell PSMA and Integrins. Int. J. Mol. Sci..

[23] Solnes L.B., Shokeen M., Pandit-Taskar N. (2021). Novel Agents and Future Perspectives on Theranostics. Semin. Radiat. Oncol..

[24] Mirshahvalad S.A., Beheshti M., Metser U., Jiang D.M., Wong R., Alrekhais I., Veit-Haibach P. (2025). Theranostic: A Primer for Radiologists. Can. Assoc. Radiol. J..

[25] Kuyumcu S., Has-Şimşek D. (2024). Evaluating the efficacy of [177Lu] Lu-FAP-2286 in combination therapy for metastatic breast cancer. Eur. J. Nucl. Med. Mol. Imaging.

[26] Zhao Y., Su X., Xiang B., Zhang S., Zhou X. (2025). Application of (68)Ga- and (177)Lu-Labeled FAP Inhibitor in Evaluation and Treatment of Cardiac Fibrosis After Myocardial Infarction. MedComm.

[27] Trujillo-Benítez D., Luna-Gutiérrez M., Aguirre-De Paz J.G., Cruz-Nova P., Bravo-Villegas G., Vargas-Ahumada J.E., Vallejo-Armenta P., Morales-Avila E., Jiménez-Mancilla N., Oros-Pantoja R. (2024). (68)Ga-DOTA-D-Alanine-BoroPro Radiotracer for Imaging of the Fibroblast Activation Protein in Malignant and Non-Malignant Diseases. Pharmaceutics.

[28] Fan M., Yao J., Zhao Z., Zhang X., Lu J. (2024). Application of 99mTc-Labeled WL12 peptides as a tumor PD-L1-Targeted SPECT Imaging Agent: Kit formulation, preclinical evaluation, and study on the influence of Coligands. Pharmaceuticals.

[29] Boschi A., Urso L., Uccelli L., Martini P., Filippi L. (2024). 99mTc-labeled FAPI compounds for cancer and inflammation: From radiochemistry to the first clinical applications. EJNMMI Radiopharm. Chem..

[30] Choi J., Beaino W., Fecek R.J., Fabian K.P.L., Laymon C.M., Kurland B.F., Storkus W.J., Anderson C.J. (2018). Combined VLA-4-Targeted Radionuclide Therapy and Immunotherapy in a Mouse Model of Melanoma. J. Nucl. Med..

[31] Rouanet J., Benboubker V., Akil H., Hennino A., Auzeloux P., Besse S., Pereira B., Delorme S., Mansard S., D’Incan M. (2020). Immune checkpoint inhibitors reverse tolerogenic mechanisms induced by melanoma targeted radionuclide therapy. Cancer Immunol. Immunother..

[32] Jiao R., Allen K.J.H., Malo M.E., Rickles D., Dadachova E. (2020). Evaluating the Combination of Radioimmunotherapy and Immunotherapy in a Melanoma Mouse Model. Int. J. Mol. Sci..

[33] Malo M.E., Allen K.J.H., Jiao R.B., Frank C., Rickles D., Dadachova E. (2020). Mechanistic Insights into Synergy between Melanin-Targeting Radioimmunotherapy and Immunotherapy in Experimental Melanoma. Int. J. Mol. Sci..

[34] Foster A., Nigam S., Tatum D.S., Raphael I., Xu J.D., Kumar R., Plakseychuk E., Latoche J.D., Vincze S., Li B. (2021). Novel theranostic agent for PET imaging and targeted radiopharmaceutical therapy of tumour-infiltrating immune cells in glioma. EBioMedicine.

[35] Ren J.Y., Xu M.X., Chen J.Y., Ding J., Wang P.P., Huo L., Li F., Liu Z.B. (2021). PET imaging facilitates antibody screening for synergistic radioimmunotherapy with a 177Lu-labeled αPD-L1 antibody. Theranostics.

[36] Vito A., Rathmann S., Mercanti N., El-Sayes N., Mossman K., Valliant J. (2021). Combined Radionuclide Therapy and Immunotherapy for Treatment of Triple Negative Breast Cancer. Int. J. Mol. Sci..

[37] Dabagian H., Taghvaee T., Martorano P., Martinez D., Samanta M., Watkins C.M., Chai R., Mansfield A., Graham T.J., Maris J.M. (2021). PARP Targeted Alpha-Particle Therapy Enhances Response to PD-1 Immune-Checkpoint Blockade in a Syngeneic Mouse Model of Glioblastoma. ACS Pharmacol. Transl. Sci..

[38] Czernin J., Current K., Mona C.E., Nyiranshuti L., Hikmat F., Radu C.G., Lückerath K. (2021). Immune-Checkpoint Blockade Enhances (225)Ac-PSMA617 Efficacy in a Mouse Model of Prostate Cancer. J. Nucl. Med..

[39] Jagodinsky J.C., Jin W.J., Bates A.M., Hernandez R., Grudzinski J.J., Marsh I.R., Chakravarty I., Arthur I.S., Zangl L.M., Brown R.J. (2021). Temporal analysis of type 1 interferon activation in tumor cells following external beam radiotherapy or targeted radionuclide therapy. Theranostics.

[40] Patel R.B., Hernandez R., Carlson P., Grudzinski J., Bates A.M., Jagodinsky J.C., Erbe A., Marsh I.R., Arthur I., Aluicio-Sarduy E. (2021). Low-dose targeted radionuclide therapy renders immunologically cold tumors responsive to immune checkpoint blockade. Sci. Transl. Med..

[41] Li M., Liu D., Lee D., Cheng Y., Baumhover N.J., Marks B.M., Sagastume E.A., Ballas Z.K., Johnson F.L., Morris Z.S. (2021). Targeted Alpha-Particle Radiotherapy and Immune Checkpoint Inhibitors Induces Cooperative Inhibition on Tumor Growth of Malignant Melanoma. Cancers.

[42] Guzik P., Siwowska K., Fang H.Y., Cohrs S., Bernhardt P., Schibli R., Müller C. (2021). Promising potential of [(177)Lu]Lu-DOTA-folate to enhance tumor response to immunotherapy-a preclinical study using a syngeneic breast cancer model. Eur. J. Nucl. Med. Mol. Imaging.

[43] Ferreira C.D., Heidari P., Ataeinia B., Sinevici N., Granito A., Kumar H.M., Wehrenberg-Klee E., Mahmood U. (2022). Immune Checkpoint Inhibitor-Mediated Cancer Theranostics with Radiolabeled Anti-Granzyme B Peptide. Pharmaceutics.

[44] Wen X., Zeng X., Cheng X., Zeng X., Liu J., Zhang Y., Li Y., Chen H., Huang J., Guo Z. (2022). PD-L1-Targeted Radionuclide Therapy Combined with αPD-L1 Antibody Immunotherapy Synergistically Improves the Antitumor Effect. Mol. Pharm..

[45] Potluri H.K., Ferreira C.A., Grudzinski J., Massey C., Aluicio-Sarduy E., Engle J.W., Kwon O., Marsh I.R., Bednarz B.P., Hernandez R. (2022). Antitumor efficacy of (90)Y-NM600 targeted radionuclide therapy and PD-1 blockade is limited by regulatory T cells in murine prostate tumors. J. Immunother. Cancer.

[46] Wen X.J., Zeng X.Y., Shi C.R., Liu J., Zhang Y.R., Shi M.Q., Li J.C., Chen H.J., Zhuang R.Q., Chen X.Y. (2023). Optimum Combination of Radiopharmaceuticals-Based Targeting-Triggering-Therapy Effect and PD-L1 Blockade Immunotherapy. Adv. Therap..

[47] Luna-Gutiérrez M., Azorín-Vega E., Oros-Pantoja R., Ocampo-García B., Cruz-Nova P., Jiménez-Mancilla N., Bravo-Villegas G., Santos-Cuevas C., Meléndez-Alafort L., Ferro-Flores G. (2025). Lutetium-177 labeled iPD-L1 as a novel immunomodulator for cancer-targeted radiotherapy. EJNMMI Radiopharm. Chem..

[48] Zhao L., Pang Y., Zhou Y., Chen J., Fu H., Guo W., Xu W., Xue X., Su G., Sun L. (2024). Antitumor efficacy and potential mechanism of FAP-targeted radioligand therapy combined with immune checkpoint blockade. Signal Transduct. Target. Ther..

[49] Kleinendorst S.C., Oosterwijk E., Molkenboer-Kuenen J., Frielink C., Franssen G.M., Boreel D.F., Tamborino G., Gloudemans M., Hendrikx M., Kroon D. (2024). Towards effective CAIX-targeted radionuclide and checkpoint inhibition combination therapy for advanced clear cell renal cell carcinoma. Theranostics.

[50] Chen J., Zhou Y., Pang Y., Fu K., Luo Q., Sun L., Wu H., Lin Q., Su G., Chen X. (2025). FAP-targeted radioligand therapy with (68)Ga/(177)Lu-DOTA-2P(FAPI)(2) enhance immunogenicity and synergize with PD-L1 inhibitors for improved antitumor efficacy. J. Immunother. Cancer.

[51] Shi J., Gao H., Wu Y., Luo C., Yang G., Luo Q., Jia B., Han C., Liu Z., Wang F. (2025). Nuclear imaging of PD-L1 expression promotes the synergistic antitumor efficacy of targeted radionuclide therapy and immune checkpoint blockade. Eur. J. Nucl. Med. Mol. Imaging.

[52] Ramonaheng K., Qebetu M., Ndlovu H., Swanepoel C., Smith L., Mdanda S., Mdlophane A., Sathekge M. (2024). Activity quantification and dosimetry in radiopharmaceutical therapy with reference to (177)Lutetium. Front. Nucl. Med..

[53] Tran-Gia J., Cicone F., Koole M., Giammarile F., Gear J., Deshayes E., Gabiña P.M., Cremonesi M., Wadsley J., Bernhardt P. (2025). Rethinking Dosimetry: A European Perspective. J. Nucl. Med..

[54] Yusufaly T., Roncali E., Brosch-Lenz J., Uribe C., Jha A.K., Currie G., Dutta J., El-Fakhri G., McMeekin H., Pandit-Taskar N. (2025). Computational Nuclear Oncology Toward Precision Radiopharmaceutical Therapies: Current Tools, Techniques, and Uncharted Territories. J. Nucl. Med..

[55] Kao Y.H. (2025). Direct Correlation of Tumor Absorbed Dose with Overall Survival in Metastatic Castration-Resistant Prostate Cancer Treated with (177)Lu Prostate-Specific Membrane Antigen. J. Nucl. Med. Technol..

[56] Abdollahi H., Yousefirizi F., Shiri I., Brosch-Lenz J., Mollaheydar E., Fele-Paranj A., Shi K., Zaidi H., Alberts I., Soltani M. (2024). Theranostic digital twins: Concept, framework and roadmap towards personalized radiopharmaceutical therapies. Theranostics.

[57] Brosch-Lenz J., Yousefirizi F., Zukotynski K., Beauregard J.M., Gaudet V., Saboury B., Rahmim A., Uribe C. (2021). Role of Artificial Intelligence in Theranostics: Toward Routine Personalized Radiopharmaceutical Therapies. PET Clin..

[58] Filippi L., Chiaravalloti A., Schillaci O., Cianni R., Bagni O. (2020). Theranostic approaches in nuclear medicine: Current status and future prospects. Expert Rev. Med. Devices.

[59] Shah A., Dabhade A., Bharadia H., Parekh P.S., Yadav M.R., Chorawala M.R. (2024). Navigating the landscape of theranostics in nuclear medicine: Current practice and future prospects. Z. Naturforsch. C J. Biosci..

[60] Strosberg J.R., Caplin M.E., Kunz P.L., Ruszniewski P.B., Bodei L., Hendifar A., Mittra E., Wolin E.M., Yao J.C., Pavel M.E. (2021). (177)Lu-Dotatate plus long-acting octreotide versus high-dose long-acting octreotide in patients with midgut neuroendocrine tumours (NETTER-1): Final overall survival and long-term safety results from an open-label, randomised, controlled, phase 3 trial. Lancet Oncol..

[61] Sitani K., Parghane R.V., Talole S., Basu S. (2021). Long-term outcome of indigenous (177)Lu-DOTATATE PRRT in patients with Metastatic Advanced Neuroendocrine Tumours: A single institutional observation in a large tertiary care setting. Br. J. Radiol..

[62] Baum R.P., Kluge A.W., Kulkarni H., Schorr-Neufing U., Niepsch K., Bitterlich N., van Echteld C.J. (2016). [(177)Lu-DOTA](0)-D-Phe(1)-Tyr(3)-Octreotide ((177)Lu-DOTATOC) For Peptide Receptor Radiotherapy in Patients with Advanced Neuroendocrine Tumours: A Phase-II Study. Theranostics.

[63] Luna-Gutiérrez M., Hernández-Ramírez R., Soto-Abundiz A., García-Pérez O., Ancira-Cortez A., López-Buenrostro S., Gibbens-Bandala B., Soldevilla-Gallardo I., Lara-Almazán N., Rojas-Pérez M. (2023). Improving Overall Survival and Quality of Life in Patients with Prostate Cancer and Neuroendocrine Tumors Using (177)Lu-iPSMA and (177)Lu-DOTATOC: Experience after 905 Treatment Doses. Pharmaceutics.

[64] Fizazi K., Herrmann K., Krause B.J., Rahbar K., Chi K.N., Morris M.J., Sartor O., Tagawa S.T., Kendi A.T., Vogelzang N. (2023). Health-related quality of life and pain outcomes with [(177)Lu]Lu-PSMA-617 plus standard of care versus standard of care in patients with metastatic castration-resistant prostate cancer (VISION): A multicentre, open-label, randomised, phase 3 trial. Lancet Oncol..

[65] Morris M.J., Castellano D., Herrmann K., de Bono J.S., Shore N.D., Chi K.N., Crosby M., Piulats J.M., Fléchon A., Wei X.X. (2024). (177)Lu-PSMA-617 versus a change of androgen receptor pathway inhibitor therapy for taxane-naive patients with progressive metastatic castration-resistant prostate cancer (PSMAfore): A phase 3, randomised, controlled trial. Lancet.

[66] Emmett L., Subramaniam S., Crumbaker M., Joshua A.M., Sandhu S., Nguyen A., Weickhardt A., Lee S.T., Ng S., Francis R.J. (2025). Overall survival and quality of life with [(177)Lu]Lu-PSMA-617 plus enzalutamide versus enzalutamide alone in metastatic castration-resistant prostate cancer (ENZA-p): Secondary outcomes from a multicentre, open-label, randomised, phase 2 trial. Lancet Oncol..

[67] Bülbül O., Ünek İ.T., Kefi A., Tuna E.B., Bekiş R. (2020). Factors affecting overall survival and progression-free survival in patients with metastatic castration resistant prostate cancer received (177)Lu PSMA I&T therapy. Hell. J. Nucl. Med..

[68] Ghosh S., and Ghosh A. (2021). Activation of DNA damage response signaling in mammalian cells by ionizing radiation. Free. Radic. Res..

[69] Salvatori M., Cremonesi M., Indovina L., Chianelli M., Pacilio M., Danieli R., Chiesa C., Zanzonico P. (2022). Radiobiology and radiation dosimetry in nuclear medicine. Nuclear Oncology: From Pathophysiology to Clinical Applications.

[70] Khazaei Monfared Y., Heidari P., Klempner S.J., Mahmood U., Parikh A.R., Hong T.S., Strickland M.R., Esfahani S.A. (2023). DNA Damage by Radiopharmaceuticals and Mechanisms of Cellular Repair. Pharmaceutics.

[71] Gerelchuluun A., Tsuboi K., Sakae T., Gerelchuluun A. (2020). DNA Damage, Repair Mechanisms, and Chromosomal Aberrations. Proton Beam Radiotherapy: Physics and Biology.

[72] Salvatori M., Cremonesi M., Indovina L., Chianelli M., McEwan A.J.B., Zanzonico P., Strauss H.W., Mariani G., Volterrani D., Larson S.M. (2013). Radiobiology and Radiation Dosimetry in Nuclear Medicine: Therapy, Diagnosis, and Considerations for Sensitive Populations. Nuclear Oncology: Pathophysiology and Clinical Applications.

[73] Fabbrizi M.R., Doggett T.J., Hughes J.R., Melia E., Dufficy E.R., Hill R.M., Goula A., Phoenix B., Parsons J.L. (2024). Inhibition of key DNA double strand break repair protein kinases enhances radiosensitivity of head and neck cancer cells to X-ray and proton irradiation. Cell Death Discov..

[74] Schipler A., Iliakis G. (2013). DNA double-strand–break complexity levels and their possible contributions to the probability for error-prone processing and repair pathway choice. Nucleic Acids Res..

[75] Bussink J., Span P.N. (2015). γ-H2AX foci in peripheral blood lymphocytes to quantify radiation-induced DNA damage after 177Lu-DOTA-Octreotate peptide receptor radionuclide therapy. J. Nucl. Med..

[76] Mavragani I.V., Nikitaki Z., Souli M.P., Aziz A., Nowsheen S., Aziz K., Rogakou E., Georgakilas A.G. (2017). Complex DNA Damage: A Route to Radiation-Induced Genomic Instability and Carcinogenesis. Cancers.

[77] Tamborino G., Nonnekens J., De Saint-Hubert M., Struelens L., Feijtel D., de Jong M., Konijnenberg M.W. (2022). Dosimetric evaluation of the effect of receptor heterogeneity on the therapeutic efficacy of peptide receptor radionuclide therapy: Correlation with DNA damage induction and in vivo survival. J. Nucl. Med..

[78] Schumann S., Scherthan H., Lapa C., Serfling S., Muhtadi R., Lassmann M., Eberlein U. (2019). DNA damage in blood leucocytes of prostate cancer patients during therapy with 177 Lu-PSMA. Eur. J. Nucl. Med. Mol. Imaging.

[79] Eberlein U., Nowak C., Bluemel C., Buck A.K., Werner R.A., Scherthan H., Lassmann M. (2015). DNA damage in blood lymphocytes in patients after 177 Lu peptide receptor radionuclide therapy. Eur. J. Nucl. Med. Mol. Imaging.

[80] Ranzani M., Hindupur S., Cicconi A., Sastre-Moreno G., Kristian A., Engstler B.S., Pinch B., Hartnagel C., Gorses D., Simon E. (2024). Abstract LB222: Elucidating the cellular responses and mechanism of action of 177Lu-based radioligand therapy. Cancer Res..

[81] Ritt P., Jobic C., Beck M., Schmidkonz C., Kuwert T., Uder M., Brand M. (2021). Dissimilar DNA damage to blood lymphocytes after 177Lu-labeled DOTATOC or prostate-specific membrane antigen therapy. J. Nucl. Med..

[82] Bertolet A., Ramos-Méndez J., Paganetti H., Schuemann J. (2021). The relation between microdosimetry and induction of direct damage to DNA by alpha particles. Phys. Med. Biol..

[83] Evans H.J. (1992). Radiation biology. Alpha-particle after effects. Nature.

[84] Göring L., Schumann S., Müller J., Buck A.K., Port M., Lassmann M., Scherthan H., Eberlein U. (2022). Repair of α-particle-induced DNA damage in peripheral blood mononuclear cells after internal ex vivo irradiation with 223Ra. Eur. J. Nucl. Med. Mol. Imaging.

[85] Bannik K., Madas B., Jarzombek M., Sutter A., Siemeister G., Mumberg D., Zitzmann-Kolbe S. (2019). Radiobiological effects of the alpha emitter Ra-223 on tumor cells. Sci. Rep..

[86] Howell R.W. (2023). Advancements in the use of Auger electrons in science and medicine during the period 2015–2019. Int. J. Radiat. Biol..

[87] Dong S., Lyu X., Yuan S., Wang S., Li W., Chen Z., Yu H., Li f., Jiang Q. (2020). Oxidative stress: A critical hint in ionizing radiation induced pyroptosis. Radiat. Med. Prot..

[88] Zheng Z., Su J., Bao X., Wang H., Bian C., Zhao Q., Jiang X. (2023). Mechanisms and applications of radiation-induced oxidative stress in regulating cancer immunotherapy. Front. Immunol..

[89] Li N., Yu L., Wang J., Gao X., Chen Y., Pan W., Tang B. (2018). A mitochondria-targeted nanoradiosensitizer activating reactive oxygen species burst for enhanced radiation therapy. Chem. Sci..

[90] Tan J., Sun X., Zhao H., Guan H., Gao S., Zhou P.K. (2023). Double-strand DNA break repair: Molecular mechanisms and therapeutic targets. MedComm.

[91] D’Andrea F.P., Safwat A., Kassem M., Gautier L., Overgaard J., Horsman M.R. (2011). Cancer stem cell overexpression of nicotinamide N-methyltransferase enhances cellular radiation resistance. Radiother. Oncol..

[92] D’Andrea F.P. (2012). Intrinsic radiation resistance of mesenchymal cancer stem cells and implications for treatment response in a murine sarcoma model. Dan. Med. J..

[93] Van Haren M.J., Zhang Y., Thijssen V., Buijs N., Gao Y., Mateuszuk L., Fedak F.A., Kij A., Campagna R., Sartini D. (2021). Macrocyclic peptides as allosteric inhibitors of nicotinamide N-methyltransferase (NNMT). RSC Chem. Biol..

[94] Gao Y., Van Haren M.J., Buijs N., Innocenti P., Zhang Y., Sartini D., Campagna R., Emanuelli M., Parsons R.B., Jespers W. (2021). Potent inhibition of nicotinamide N-methyltransferase by alkene-linked bisubstrate mimics bearing electron deficient aromatics. J. Med. Chem..

[95] van Haren M.J., Gao Y., Buijs N., Campagna R., Sartini D., Emanuelli M., Mateuszuk L., Kij A., Chlopicki S., Escudé Martinez de Castilla P. (2021). Esterase-sensitive prodrugs of a potent bisubstrate inhibitor of nicotinamide N-methyltransferase (NNMT) display cellular activity. Biomolecules.

[96] Dent P., Yacoub A., Contessa J., Caron R., Amorino G., Valerie K., Hagan M.P., Grant S., Schmidt-Ullrich R. (2003). Stress and radiation-induced activation of multiple intracellular signaling pathways. Radiat. Res..

[97] Qin X., Jiang H., Liu Y., Zhang H., Tian M. (2022). Radionuclide imaging of apoptosis for clinical application. Eur. J. Nucl. Med. Mol. Imaging.

[98] Lundsten S., Spiegelberg D., Stenerlöw B., Nestor M. (2019). The HSP90 inhibitor onalespib potentiates 177Lu-DOTATATE therapy in neuroendocrine tumor cells. Int. J. Oncol..

[99] Lundsten S., Berglund H., Jha P., Krona C., Hariri M., Nelander S., Lane D.P., Nestor M. (2021). p53-Mediated Radiosensitization of 177Lu-DOTATATE in Neuroblastoma Tumor Spheroids. Biomolecules.

[100] Yeh M.C., Tse B.W.C., Fletcher N.L., Houston Z.H., Lund M., Volpert M., Stewart C., Sokolowski K.A., Jeet V., Thurecht K.J. (2020). Targeted beta therapy of prostate cancer with (177)Lu-labelled Miltuximab® antibody against glypican-1 (GPC-1). EJNMMI Res..

[101] Yong K.J., Milenic D.E., Baidoo K.E., Brechbiel M.W. (2016). Mechanisms of cell killing response from low linear energy transfer (LET) radiation originating from 177Lu radioimmunotherapy targeting disseminated intraperitoneal tumor xenografts. Int. J. Mol. Sci..

[102] Hernandez M.C., Knox S.J. (2003). Radiobiology of radioimmunotherapy with 90Y ibritumomab tiuxetan (Zevalin). Seminars in Oncology.

[103] Suominen M.I., Fagerlund K.M., Rissanen J.P., Konkol Y.M., Morko J.P., Peng Z., Alhoniemi E.J., Laine S.K., Corey E., Mumberg D. (2017). Radium-223 inhibits osseous prostate cancer growth by dual targeting of cancer cells and bone microenvironment in mouse models. Clin. Cancer Res..

[104] Singh Jaggi J., Henke E., Seshan S.V., Kappel B.J., Chattopadhyay D., May C., McDevitt M.R., Nolan D., Mittal V., Benezra R. (2007). Selective alpha-particle mediated depletion of tumor vasculature with vascular normalization. PLoS ONE.

[105] Kumar P., Koach J., Nekritz E., Mukherjee S., Braun B.S., DuBois S.G., Nasholm N., Haas-Kogan D., Matthay K.K., Weiss W.A. (2024). Aurora Kinase A inhibition enhances DNA damage and tumor cell death with (131)I-MIBG therapy in high-risk neuroblastoma. EJNMMI Res..

[106] Nayak T.K., Norenberg J.P., Anderson T.L., Prossnitz E.R., Stabin M.G., Atcher R.W. (2007). Somatostatin-receptor-targeted alpha-emitting 213Bi is therapeutically more effective than beta(-)-emitting 177Lu in human pancreatic adenocarcinoma cells. Nucl. Med. Biol..

[107] Park S.-Y., Kim J.-Y., Jun Y., Nam J.-S. (2018). Strategies to tackle radiation resistance by penetrating cancer stem cell line of scrimmage. Recent Pat. Anti-Cancer Drug Discov..

[108] O’Connor M.J. (2015). Targeting the DNA damage response in cancer. Mol. Cell.

[109] Wang C., Chen L., Fu D., Liu W., Puri A., Kellis M., Yang J. (2024). Antigen presenting cells in cancer immunity and mediation of immune checkpoint blockade. Clin. Exp. Metastasis.

[110] Ascic E., Pereira C.F. (2025). Transcription factor-mediated reprogramming to antigen-presenting cells. Curr. Opin. Genet. Dev..

[111] Varol C., Mildner A., Jung S. (2015). Macrophages: Development and tissue specialization. Annu. Rev. Immunol..

[112] Franken L., Schiwon M., Kurts C. (2016). Macrophages: Sentinels and regulators of the immune system. Cell Microbiol..

[113] Zagorulya M., Duong E., Spranger S. (2020). Impact of anatomic site on antigen-presenting cells in cancer. J. Immunother. Cancer.

[114] Marciscano A.E., Anandasabapathy N. (2021). The role of dendritic cells in cancer and anti-tumor immunity. Semin. Immunol..

[115] Jarosz-Biej M., Smolarczyk R., Cichoń T., Kułach N. (2019). Tumor Microenvironment as A "Game Changer" in Cancer Radiotherapy. Int. J. Mol. Sci..

[116] Wu Y., Pfeifer A.K., Myschetzky R., Garbyal R.S., Rasmussen P., Knigge U., Bzorek M., Kristensen M.H., Kjaer A. (2013). Induction of Anti-Tumor Immune Responses by Peptide Receptor Radionuclide Therapy with (177)Lu-DOTATATE in a Murine Model of a Human Neuroendocrine Tumor. Diagnostics.

[117] Guagnano V., Kauffmann A., Wöhrle S., Stamm C., Ito M., Barys L., Pornon A., Yao Y., Li F., Zhang Y. (2012). FGFR genetic alterations predict for sensitivity to NVP-BGJ398, a selective pan-FGFR inhibitor. Cancer Discov..

[118] Dar T.B., Henson R.M., Shiao S.L. (2018). Targeting Innate Immunity to Enhance the Efficacy of Radiation Therapy. Front. Immunol..

[119] Herrera F.G., Ronet C., Ochoa de Olza M., Barras D., Crespo I., Andreatta M., Corria-Osorio J., Spill A., Benedetti F., Genolet R. (2022). Low-Dose Radiotherapy Reverses Tumor Immune Desertification and Resistance to Immunotherapy. Cancer Discov..

[120] Liu C., Li X., Huang Q., Zhang M., Lei T., Wang F., Zou W., Huang R., Hu X., Wang C. (2023). Single-cell RNA-sequencing reveals radiochemotherapy-induced innate immune activation and MHC-II upregulation in cervical cancer. Signal Transduct. Target. Ther..

[121] Zagorulya M., Yim L., Morgan D.M., Edwards A., Torres-Mejia E., Momin N., McCreery C.V., Zamora I.L., Horton B.L., Fox J.G. (2023). Tissue-specific abundance of interferon-gamma drives regulatory T cells to restrain DC1-mediated priming of cytotoxic T cells against lung cancer. Immunity.

[122] Hernandez R., Walker K.L., Grudzinski J.J., Aluicio-Sarduy E., Patel R., Zahm C.D., Pinchuk A.N., Massey C.F., Bitton A.N., Brown R.J. (2019). (90)Y-NM600 targeted radionuclide therapy induces immunologic memory in syngeneic models of T-cell Non-Hodgkin’s Lymphoma. Commun. Biol..

[123] Malamas A.S., Gameiro S.R., Knudson K.M., Hodge J.W. (2016). Sublethal exposure to alpha radiation (223Ra dichloride) enhances various carcinomas’ sensitivity to lysis by antigen-specific cytotoxic T lymphocytes through calreticulin-mediated immunogenic modulation. Oncotarget.

[124] Park J., Hsueh P.C., Li Z., Ho P.C. (2023). Microenvironment-driven metabolic adaptations guiding CD8(+) T cell anti-tumor immunity. Immunity.

[125] Shi Z., Hu C., Zheng X., Sun C., Li Q. (2024). Feedback loop between hypoxia and energy metabolic reprogramming aggravates the radioresistance of cancer cells. Exp. Hematol. Oncol..

[126] Kumar P., Elsaidi H.R., Zorniak B., Laurens E., Yang J., Bacchu V., Wang M., Wiebe L.I. (2016). Synthesis and Biological Evaluation of Iodoglucoazomycin (I-GAZ), an Azomycin-Glucose Adduct with Putative Applications in Diagnostic Imaging and Radiotherapy of Hypoxic Tumors. ChemMedChem.

[127] Daguenet E., Louati S., Wozny A.S., Vial N., Gras M., Guy J.B., Vallard A., Rodriguez-Lafrasse C., Magné N. (2020). Radiation-induced bystander and abscopal effects: Important lessons from preclinical models. Br. J. Cancer..

[128] Matarèse B.F.E., Rusin A., Seymour C., Mothersill C. (2023). Quantum Biology and the Potential Role of Entanglement and Tunneling in Non-Targeted Effects of Ionizing Radiation: A Review and Proposed Model. Int. J. Mol. Sci..

[129] Zhang D., Zhou T., He F., Rong Y., Lee S.H., Wu S., Zuo L. (2016). Reactive oxygen species formation and bystander effects in gradient irradiation on human breast cancer cells. Oncotarget.

[130] Bishayee A., Hill H.Z., Stein D., Rao D.V., Howell R.W. (2001). Free radical-initiated and gap junction-mediated bystander effect due to nonuniform distribution of incorporated radioactivity in a three-dimensional tissue culture model. Radiat. Res..

[131] Xue L.Y., Butler N.J., Makrigiorgos G.M., Adelstein S.J., Kassis A.I. (2002). Bystander effect produced by radiolabeled tumor cells in vivo. Proc. Natl. Acad. Sci. USA.

[132] Boyd M., Ross S.C., Dorrens J., Fullerton N.E., Tan K.W., Zalutsky M.R., Mairs R.J. (2006). Radiation-induced biologic bystander effect elicited in vitro by targeted radiopharmaceuticals labeled with alpha-, beta-, and auger electron-emitting radionuclides. J. Nucl. Med..

[133] Ye F., Ning J., Liu X., Jin X., Wang T., Li Q. (2016). The influence of non-DNA-targeted effects on carbon ion-induced low-dose hyper-radiosensitivity in MRC-5 cells. J. Radiat. Res..

[134] Joiner M.C., Marples B., Lambin P., Short S.C., Turesson I. (2001). Low-dose hypersensitivity: Current status and possible mechanisms. Int. J. Radiat. Oncol. Biol. Phys..

[135] Kumar C., Shetake N., Desai S., Kumar A., Samuel G., Pandey B.N. (2016). Relevance of radiobiological concepts in radionuclide therapy of cancer. Int. J. Radiat. Biol..

[136] Morris Z.S., Wang A.Z., Knox S.J. (2021). The Radiobiology of Radiopharmaceuticals. Semin. Radiat. Oncol..

[137] Janopaul-Naylor J.R., Shen Y., Qian D.C., Buchwald Z.S. (2021). The Abscopal Effect: A Review of Pre-Clinical and Clinical Advances. Int. J. Mol. Sci..

[138] Miranda S., Correia M., Dias A.G., Pestana A., Soares P., Nunes J., Lima J., Máximo V., Boaventura P. (2020). Evaluation of the role of mitochondria in the non-targeted effects of ionizing radiation using cybrid cellular models. Sci. Rep..

[139] Falahat R., Berglund A., Putney R.M., Perez-Villarroel P., Aoyama S., Pilon-Thomas S., Barber G.N., Mulé J.J. (2021). Epigenetic reprogramming of tumor cell-intrinsic STING function sculpts antigenicity and T cell recognition of melanoma. Proc. Natl. Acad. Sci. USA.

[140] Amaresan R., Gopal U. (2023). Cell surface GRP78: A potential mechanism of therapeutic resistant tumors. Cancer Cell Int..

[141] Jia Y., Jia R., Dai Z., Zhou J., Ruan J., Chng W., Cai Z., Zhang X. (2024). Stress granules in cancer: Adaptive dynamics and therapeutic implications. iScience.

[142] Swati, Chadha V.D. (2021). Role of epigenetic mechanisms in propagating off-targeted effects following radiation based therapies—A review. Mutat. Res.-Rev. Mutat. Res..

[143] Chen Y.F., Luh F., Ho Y.S., Yen Y. (2024). Exosomes: A review of biologic function, diagnostic and targeted therapy applications, and clinical trials. J. Biomed. Sci..

[144] Coleman C.N., Eke I., Makinde A.Y., Chopra S., Demaria S., Formenti S.C., Martello S., Bylicky M., Mitchell J.B., Aryankalayil M.J. (2020). Radiation-induced Adaptive Response: New Potential for Cancer Treatment. Clin. Cancer Res..

[145] Nikitaki Z., Nikolov V., Mavragani I.V., Mladenov E., Mangelis A., Laskaratou D.A., Fragkoulis G.I., Hellweg C.E., Martin O.A., Emfietzoglou D. (2016). Measurement of complex DNA damage induction and repair in human cellular systems after exposure to ionizing radiations of varying linear energy transfer (LET). Free Radic. Res..

[146] Lindholm C., Acheva A., Salomaa S. (2010). Clastogenic plasma factors: A short overview. Radiat. Env. Biophys..

[147] Murray I., Du Y. (2021). Systemic Radiotherapy of Bone Metastases With Radionuclides. Clin. Oncol..

[148] Jin J., Wu X., Yin J., Li M., Shen J., Li J., Zhao Y., Zhao Q., Wu J., Wen Q. (2019). Identification of Genetic Mutations in Cancer: Challenge and Opportunity in the New Era of Targeted Therapy. Front. Oncol..

[149] Teuter M., Hu Y., Ross T.L., Lolatte K., Ott M., Bengel F.M., Balakrishnan A., Bankstahl J.P. (2025). Longitudinal multi-tracer imaging of hepatocellular carcinoma identifies novel stage- and oncogene-specific changes. Nucl. Med. Biol..

